# Growth Hormone Upregulates Mediators of Melanoma Drug Efflux and Epithelial-to-Mesenchymal Transition In Vitro and In Vivo

**DOI:** 10.3390/cancers12123640

**Published:** 2020-12-04

**Authors:** Yanrong Qian, Reetobrata Basu, Samuel C. Mathes, Nathan A. Arnett, Silvana Duran-Ortiz, Kevin R. Funk, Alison L. Brittain, Prateek Kulkarni, Joseph C. Terry, Emily Davis, Jordyn T. Singerman, Brooke E. Henry, Edward O. List, Darlene E. Berryman, John J. Kopchick

**Affiliations:** 1 Edison Biotechnology Institute, Ohio University, Athens, OH 45701, USA; qiany@ohio.edu (Y.Q.); basu@ohio.edu (R.B.); mathes@ohio.edu (S.C.M.); na366913@ohio.edu (N.A.A.); sd504111@ohio.edu (S.D.-O.); funkk@ohio.edu (K.R.F.); ab647713@ohio.edu (A.L.B.); pk585316@ohio.edu (P.K.); jt321916@ohio.edu (J.C.T.); ed218514@ohio.edu (E.D.); js243112@ohio.edu (J.T.S.); bh997614@ohio.edu (B.E.H.); list@ohio.edu (E.O.L.); berrymad@ohio.edu (D.E.B.); 2 Department of Biological Sciences, Ohio University, Athens, OH 45701, USA; 3 Russ College of Engineering, Ohio University, Athens, OH 45701, USA; 4 Molecular Cellular Biology Program, Ohio University, Athens, OH 45701, USA; 5 Department of Biomedical Sciences, Heritage College of Osteopathic Medicine, Ohio University, Athens, OH 45701, USA

**Keywords:** growth hormone, growth hormone receptor, insulin-like growth factor-1, melanoma, multidrug efflux pumps, epithelial-to-mesenchymal transition

## Abstract

**Simple Summary:**

Growth hormone (GH) action is strongly implicated in the progression and therapy resistance in several types of solid tumors which overexpress the GH receptor (GHR). The aim of our study was to characterize the effects of GH and its downstream effector insulin-like growth factor 1 (IGF-1) on melanoma using in vitro and in vivo models. We confirmed an IGF-1-independent role of elevated circulating GH in upregulating key mechanisms of therapy resistance and malignancy with analyses conducted at the molecular and cellular level. We identified that GH upregulates key mechanisms of therapy resistance and metastases in melanoma tumors in an IGF-1 dependent and independent manner by upregulating multidrug efflux pumps and EMT transcription factors. Our study reveals that GH action renders an intrinsic drug resistance phenotype to the melanoma tumors—a clinically crucial property of GH verifiable in other human cancers with GHR expression.

**Abstract:**

Growth hormone (GH) and the GH receptor (GHR) are expressed in a wide range of malignant tumors including melanoma. However, the effect of GH/insulin-like growth factor (IGF) on melanoma in vivo has not yet been elucidated. Here we assessed the physical and molecular effects of GH on mouse melanoma B16-F10 and human melanoma SK-MEL-30 cells in vitro. We then corroborated these observations with syngeneic B16-F10 tumors in two mouse lines with different levels of GH/IGF: bovine GH transgenic mice (bGH; high GH, high IGF-1) and GHR gene-disrupted or knockout mice (GHRKO; high GH, low IGF-1). In vitro, GH treatment enhanced mouse and human melanoma cell growth, drug retention and cell invasion. While the in vivo tumor size was unaffected in both bGH and GHRKO mouse lines, multiple drug-efflux pumps were up regulated. This intrinsic capacity of therapy resistance appears to be GH dependent. Additionally, epithelial-to-mesenchymal transition (EMT) gene transcription markers were significantly upregulated in vivo supporting our current and recent in vitro observations. These syngeneic mouse melanoma models of differential GH/IGF action can be valuable tools in screening for therapeutic options where lowering GH/IGF-1 action is important.

## 1. Introduction

Accumulating evidence implicates growth hormone (GH) in the development and progression of a wide range of malignancies [1,2,3,4,5,6,7], including breast [8,9,10,11,12,13,14,15], prostate [16,17], colorectal [18,19], glioma [20], pancreatic [21], thyroid [22], liver [23] and endometrial cancers [24,25]. GH is a peptide hormone produced by pituitary somatotrophs and acts on multiple tissues including bone, muscle, fat, liver, pancreas, heart, thymus and kidney. GH is anabolic in bones and muscle, while catabolic in adipose tissue, and exerts distinct metabolic effects on liver, muscle, and fat throughout the lifespan of an individual [26]. GH’s actions are mediated first through binding to the preformed GHR dimer, which activates intracellular signal transduction pathways known to be critical for GH’s multiple effects [27,28]. These pathways include the Janus kinase-2 (JAK-2) and signal transducers and activators of transcription 5 (STAT5), STAT1 and STAT3, the c-Src mediated p44/42 mitogen-activated protein kinase (ERK or MAPK), and the phosphoinositide 3-kinase (PI3K) pathways [27,28,29,30]. Upregulation of selected components of these pathways has been observed in a wide range of malignant GH-responsive tumors, supporting the involvement of GH-induced GHR activation in tumor growth.

IGF-1 is another potent growth factor, which has autocrine, paracrine and endocrine effects and is an important drug target in cancer [31,32,33]. GH induces the majority of circulating IGF-1 production primarily in the liver as well as in several other tissues. Both GH and IGF-1 are important components of GH-induced intracellular signaling pathways, and together constitute the GH/IGF-1 axis. In terms of growth, GH and IGF-1 have both unique and overlapping actions [34,35]. It is of particular interest to be able to resolve IGF-dependent and -independent effects of GH related to cancer incidence and progression. Mouse models with a dysregulated GH/IGF-1 axis—bGH (bovine GH transgenic) and GHRKO (GHR knock out)—are valuable for studying these differential effects. bGH mice have high GH (>600 ng/mL; ~400-fold increase) and high circulating IGF-1 (~500 ng/mL; ~2-fold increase) [36,37]. In contrast, GHRKO mice have a germline inactivating mutation in the GHR. These mice are small, with low levels of IGF-1 (~50 ng/mL; ~11-fold decrease), but high levels of GH (~120 ng/mL; ~12-fold increase), and are thus GH insensitive [38]. Utilization of these two mouse lines allows for a differential evaluation of the effects of elevated GH versus IGF-1 on cancers in vivo [37,38].

Several recent reviews have highlighted the profile of GH action in multiple types of cancer [6,7,39,40]. Lobie and colleagues have reported that GH expression in tumors predicts a worse survival outcome in patients with mammary and endometrial carcinoma [41]. Melmed et al. have demonstrated through a series of elegant studies that GH induces DNA damage and abrogates DNA damage repair (DDR) in normal colon tissues, alongside decreasing p53 expression by suppressing ATM kinases [42,43,44]. These actions of GH increase colon polyps and potentially direct oncogenesis and cancer progression in the colon; and indeed colon cancer incidence rates were found upregulated by several studies on acromegaly patients [22,45]. Furthermore, autocrine human (h) GH promotes oncogenicity, cell growth, survival, migration, invasion, and induces tumor angiogenesis and resistance to radiation treatment in mammary, endometrial and hepatocellular carcinomas [3,9,11,15,24,25,46,47,48,49,50,51,52,53,54].

Melanoma is one of several skin cancers and develops from transformation of the pigment-containing cells known as melanocytes. It represents less than one percent of cases but accounts for the most deaths among skin cancers [55]. The incidence of melanoma has increased over the past four decades, resulting in an intensified need to understand the mechanisms of melanoma development, to improve the effectiveness of available treatments, and to develop efficient and novel anticancer therapies.

To date, multiple in vitro studies conducted by our group and others have contributed towards the overall understanding of the effects of the GH/IGF axis on melanoma cells. We have previously shown that among nine types of human cancers in the National Cancer Institute (NCI-60) panel of 60 cell lines, all of the melanoma cell lines exhibit the highest levels of GHR, and the treatment of these cells with recombinant hGH resulted in increased melanoma proliferation and activation of oncogenic signaling intermediates [56]. We have also shown that siRNA-mediated GHR knockdown in human melanoma cells attenuated tumor progression, epithelial-to-mesenchymal transition (EMT) [57] and sensitized these cells to chemotherapy by attenuating expression of ATP-binding cassette (ABC) multidrug efflux pumps [58]. We further reported that GH upregulates the expression and activity of melanocyte-inducing transcription factor (MITF) via JAK2-STAT5 and SRC signaling in human melanoma cells, thereby inducing drug sequestration in melanosomes [59]. The ability of IGF-1 to enhance metastasis of melanoma cells through upregulation of EMT has also been reported [60]. However, to date, in vivo validation of the role of GH in melanoma is absent.

As an extension of our in vitro studies, we sought to study the effects of GH/IGF-1 on melanoma progression in vivo. B16-F10 mouse melanoma cells are widely used and well-studied [61]. The B16-F10 cell line was originally derived from C57BL/6J mice; the same background strain of both bGH transgenic and GHRKO mice. By inoculating this cell line into bGH and GHRKO mice, we were able to test the hitherto unknown role of GH and/or IGF-1 in melanoma progression in vivo.

## 2. Results

### 2.1. Melanoma Cells Are Responsive to GH Treatment

**Figure 1:**
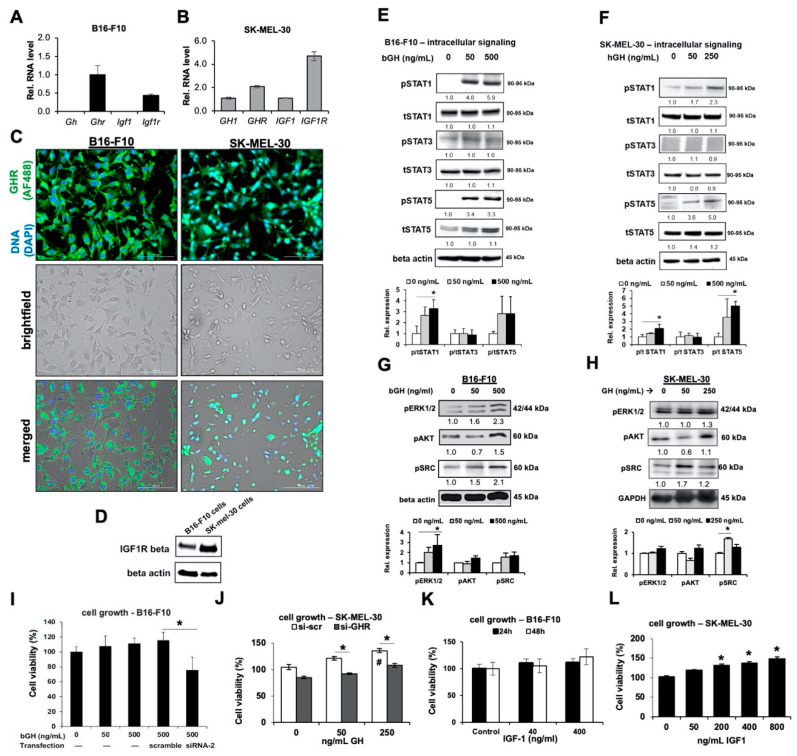
Melanoma cells are responsive to GH treatment in vitro. (**A**) Relative RNA expression was quantified for *Gh*, *Ghr*, *Igf1* and *Igf1r* genes in B16-F10 mouse melanoma cells (n = 3). (**B**) Relative RNA expression was quantified for *GH*, *GHR*, *IGF1* and *IGF1R* genes in SK-MEL-30 human melanoma cells, normalized against *GAPDH* (n = 3). (**C**) B16-F10 and SK-MEL-30 cells were stained with GHR antibody. Top panels present cellular DNA stained with DAPI (blue) and fluorescent signals from AF488-tagged anti-GHR antibody (green). Middle panels represent brightfield views while bottom panels represent merged views (20× magnification; scale bar represents 100 μm). (**D**) Expression of IGF1R-beta was detected in B16-F10 and SK-MEL-30 cells via western blotting. (**E**) Representative images of western blot analyses of phosphorylation (p) and total (t) levels of STATs 1, 3 and 5 in bovine GH (bGH) treated B16-F10 melanoma cell lysates. Cells were treated for 30 min and lysed. Densitometry analysis was performed and normalized against β-actin. The fold changes relative to control are labeled under each band. The ratio of p/t protein levels are quantified against controls and shown in bar graphs (n = 3). (**F**) Similar results for SK-MEL-30 cells treated with human (h) GH for 30 min were obtained. (**G**,**H**) Changes in GH signaling proteins, pERK1/2, pAKT and pSRC, in B16-F10 cells and SK-MEL-30 cells treated with bGH or hGH for 48 h. Protein levels were normalized against β-actin or GAPDH. (**I**) Changes in cell proliferation of B16-F10 cells transfected with scramble or GHR-targeted siRNA (siRNA-2), after 48-h treatment with bGH. (**J**) Similar results were observed in SK-MEL-30 cells transfected with scramble (si-scr) or GHR-targeted siRNA (si-GHR), after 48-h treatment with hGH (* as compared with si-scr group, # as compared with no treatment group). (**K**) Changes in cell proliferation of B16-F10 cells treated with increasing doses of IGF-1 for 24 and 48 h. (**L**) Changes in cell proliferation of SK-MEL-30 after 48-h treatment with recombinant human IGF-1 (n = 5). Data are presented as mean ± standard deviation (*, *p* < 0.05, Mann-Whitney U test).

Using realtime RT-qPCR, immunocytochemistry and western blotting, we evaluated RNA and protein expression levels of GH and GHR, as well as IGF-1 and IGF-1R in human and mouse melanoma cell lines. Both B16-F10 mouse melanoma and SK-MEL-30 human melanoma cells express *Ghr* and *Igf-1r* RNA, while SK-MEL-30 also expressed *GH1* and *IGF-1* transcripts (Figure 1A,B). Further, immunostaining with GHR-specific antibody revealed an abundant distribution of GHR on both B16-F10 and SK-MEL-30 cells (Figure 1C), while western-blot analysis revealed robust expression of IGF-1R in both cell lines (Figure 1D and Figure S9). In fact, the GHR expression on the melanoma cell lines was markedly higher than the same in non-transformed skin fibroblasts (Figure S1A). B16-F10 cells did not express detectable levels of *Gh* or *Igf-1* RNA, indicating perhaps a greater dependence on either circulating or paracrine GH or IGF-1 from the tumor microenvironment, rather than autocrine production. Even following 48 h of GH treatment, the supernatants of GH-treated B16-F10 cells showed no increase in IGF-1 protein levels (Figure S1E). GHR activation, following the addition of GH, can be evaluated by testing the phosphorylation states of signature GH-induced signaling intermediates, e.g., STAT5 as well as STATs 1 and 3, SRC, ERK1/2 (p42/44 MAPK), and AKT [27,28]. These signaling proteins have been shown to contribute to tumor proliferation and survival [28,62]. We observed a GH-induced dose-dependent increase in the phosphorylation of STATs 1 and 5, in both B16-F10 and SK-MEL-30 cells in vitro, while STAT3 phosphorylation was not affected (Figure 1E,F and Figure S9). This was distinct within 15 min of GH treatment in B16-F10 mouse melanoma cells (Figures S1B and S9), consistent with our previous observations in the following human melanoma cells: SK-MEL-5, SK-MEL-28, MALME-3M, and MDA-MB-435 [57,58]. Additionally, 500 ng/mL bGH treatment in B16-F10 cells and 250 ng/mL hGH treatment in SK-MEL-30 cells, respectively, upregulated phosphorylation states of ERK1/2, AKT, and SRC (Figure 1G,H and Figure S9). Furthermore, to assess a physiological effect of up regulated GHR and IGF1-R and active GH signaling, we assessed cell proliferation in presence of extended GH treatments or following siRNA mediated GHR knockdown. High doses of exogenous GH did not significantly change the growth-rate in B16-F10 cells (Figure 1I), although 250 ng/mL GH did significantly increase SK-MEL-30 cell growth over 48 h (Figure 1J). However, in both B16-F10 and SK-MEL-30 cells, there was a consistent and significant reduction in cell growth following GHR knockdown (Figure 1I,J). 

These findings collectively indicate that both mouse and human melanoma cells express functional GHRs that are responsive to GH, shown by increased GH-induced signaling intermediates and melanoma growth in vitro. As both B16-F10 and SK-MEL-30 cells express high levels of IGF-1R, we assessed IGF-1 dose-dependent cell growth up to 48 h after treatment. We saw no significant increase in B16-F10 growth by up to 400 ng/mL IGF-1 treatment (Figure 1K), although above 200 ng/mL, IGF-1 significantly upregulated SK-MEL-30 growth in vitro (Figure 1L). Thus, the B16-F10 differed from the human SK-MEL-30 cells in the lack of autocrine GH or IGF-1 production but were alike in displaying abrogated cell growth following GHR blockade (Figure 1I). This along with the active and consistent intracellular signaling in both cell lines indicates that GH may contributes to some other intrinsic cellular phenomenon in melanoma cells, distinct from mitotic proliferation. We proceeded to evaluate the effect of the GH/IGF-1 axis in vivo in mouse syngeneic model for melanoma in C57BL/6J male and female mice transgenic for bovine GH (bGH mice) and in mice insensitive to GH action due to a dysfunctional GHR ‘knock-out’ (GHRKO mice).

### 2.2. In Syngeneic Mouse Melanoma Models, Elevated GH or IGF-1 Did Not Affect Growth Rate of B16-F10 Tumors In Vivo

**Figure 2:**
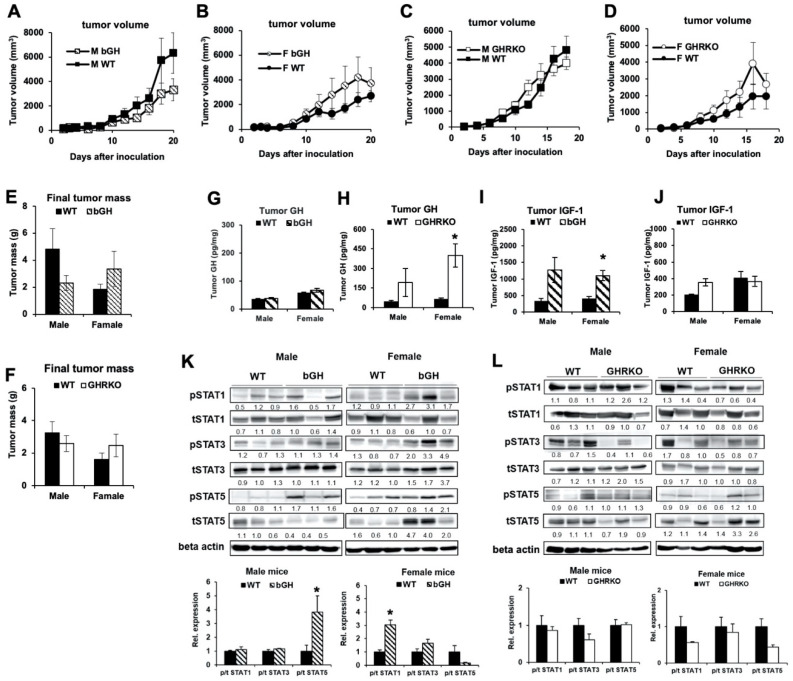
Subcutaneous B16-F10 mouse melanoma growth in bGH and GHRKO syngeneic mice. (**A**) Subcutaneous B16-F10 tumor growth in male bGH (n = 5) vs. WT mice (n = 6). (**B**) Subcutaneous B16-F10 tumor growth in female bGH (n = 7) and WT mice (n = 6). (**C**) Subcutaneous B16-F10 tumor growth in male GHRKO (n = 6) vs. WT (n = 8) mice. (**D**) Subcutaneous B16-F10 tumor growth in female GHRKO (n = 4) and WT mice (n = 5). Tumor sizes were analyzed by repeated measures (SPSS). (**E**) Weight of subcutaneous B16-F10 tumors from bGH vs. WT mice at dissection. (**F**) Weight of subcutaneous B16-F10 tumors from GHRKO vs. WT mice at dissection. (**G**) GH levels were measured in protein lysates isolated from tumors of bGH and WT mice using ELISA and normalized to total protein concentrations (n = 4). (**H**) Similar GH measurements were performed in protein lysates isolated from tumors of GHRKO and WT mice (males n = 3, females n = 4). (**I**) IGF-1 levels were measured in protein lysates isolated from tumors of bGH and WT mice using ELISA and normalized to total protein concentrations (n = 4). (**J**) Similar IGF-1 measurements were performed in protein lysates isolated from tumors of GHRKO and WT mice (males n = 3, females n = 4). (**K**) Representative images of western blot analysis of phosphorylation (p) and total (t) levels of STAT1, STAT3 and STAT5 in protein lysates isolated from tumors of bGH and WT mice. Densitometry analysis was performed and normalized against β-Actin. The relative expression levels (fold change relative to WT) are labeled under each band. The ratio of phosphorylated vs. total protein levels in tumors from bGH and WT mice are presented in bar graphs (n = 4). (**L**) Representative images of western blot analysis of phosphorylation and total levels of STAT1, STAT3, and STAT5 in protein lysates isolated from tumors of GHRKO and WT mice. (males n = 3, females n = 4). Data are presented as mean ± standard errors (*, *p* < 0.05, unpaired student’s *t*-test).

The syngeneic mouse melanoma models are unique as the bGH mice present supraphysiological GH and IGF-1 levels; while the GHRKO mice present supraphysiological GH but highly suppressed IGF-1 levels [36,37,38]. Results derived from these mice, when compared to their WT counterparts with physiological levels of GH and IGF-1, allow us to tease out the independent or perhaps synergistic effect of GH and IGF-1 on the inoculated mouse melanomas. Accordingly, the bGH and GHRKO mice and their WT counterparts were subcutaneously inoculated with B16-F10 cells, and the size of the resulting tumors were measured over time. We did not observe a significant difference in tumor volumes (Figure 2A–D) or weights (Figure 2E,F), in either the bGH (Figure 2A,B,E) or the GHRKO (Figure 2C,D,F) mice relative to controls. However, we did observe a non-significant trend towards elevated tumor size and weight specifically in female bGH (Figure 2B,E) and GHRKO (Figure 2D,F) mice compared to controls. In vivo, the implanted tumors were under the effect of both endocrine and autocrine/paracrine GH and IGF-1. Therefore, next we assessed the tumoral production of GH and IGF-1. There was no significant difference in the tumoral transcript levels of *Ghr*, *Igf-1*, and *Igf-1r* between the mice (Figure S2). Contrary to a significantly low level of *Gh* and *Igf-1* transcript levels in vitro (Figure 1A), we detected GH and IGF-1 proteins in the tumor lysates in vivo. Interestingly, there was no difference in tumoral GH or IGF-1 levels in male bGH and GHRKO vs. corresponding WT mice (Figure 2G–J). However, female mice presented significantly higher autocrine IGF-1 levels in bGH mice compared to WT controls (Figure 2I) and significantly higher autocrine GH levels in the GHRKO vs. WT mice (Figure 2H). Furthermore, we assessed the extent of GH induced intracellular signaling in the isolated tumors from bGH and GHRKO and their corresponding WT mice. 

We did observe a significantly higher level of phosphorylation of STAT5 in the male bGH vs. the WT mice (Figure 2K and Figure S9) and a significantly higher phosphorylation of STAT1 in the tumors in the female bGH vs. the WT mice (Figure 2K and Figure S9); while the GHRKO vs. WT mice did not reveal a significant difference in STAT signaling (Figure 2L and Figure S9).

GH is also known to affect glucose metabolism in various tissues [63]. Thus, we tested glycolysis and oxidative phosphorylation in these tumors. The results suggested that neither was affected by elevated GH action in vivo, similar to in vitro observations, at least at the GH concentrations tested (Figure S3). Therefore, we proceeded to inquire into the effects of GH and GHR in driving intrinsic drug resistance by upregulating ABC-cassette containing multidrug transporters [58] and EMT mediators [57], as we recently described in human melanoma cells in vitro.

### 2.3. Elevated GH Drives Expression of ATP-Binding Cassette (ABC-Type) Multidrug Efflux Pumps in Melanoma Tumors In Vitro and In Vivo

**Figure 3:**
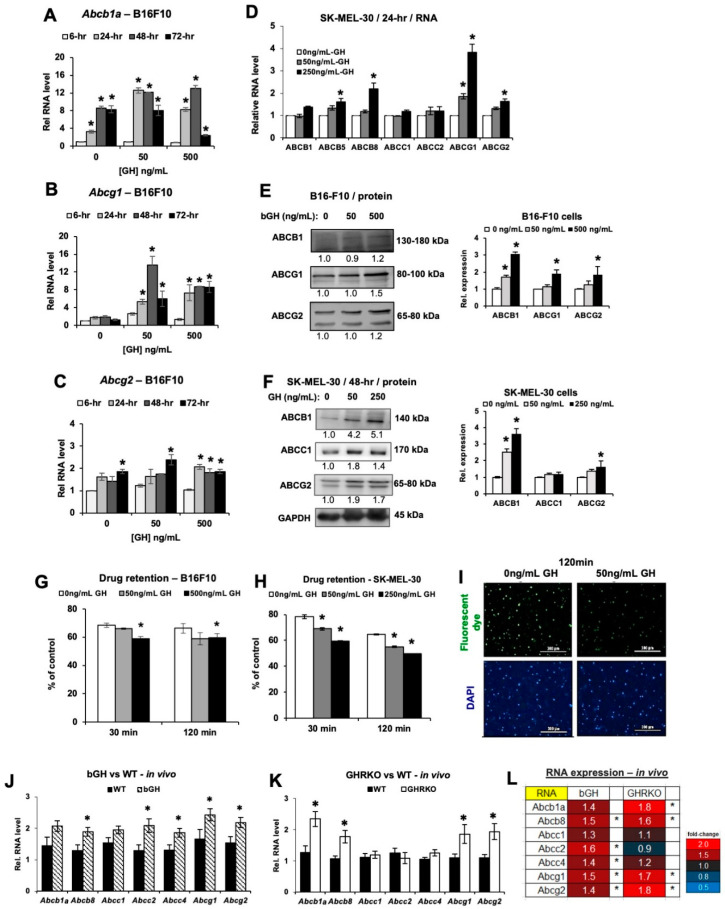
Changes in ABC efflux pumps in B16-F10 mouse melanoma in vitro and in vivo. (**A**–**C**) Changes in RNA levels for *Abcb1a*, *Abcg1*, *Abcg2* in B16-F10 mouse melanoma cells treated with bGH, normalized against reference genes (n = 3). (**D**) Changes in RNA levels for several ABC efflux pumps in SK-MEL-30 human melanoma cells treated with hGH for 24 h, normalized against *GAPDH* gene (n = 3). (**E**) Changes in proteins levels of several ABC efflux pumps of B16-F10 cells treated with bGH for 72 h. Densitometry analysis was performed and normalized to total protein (n = 3). (**F**) Changes in proteins levels of several ABC efflux pumps of SK-MEL-30 cells treated with hGH for 48 h, normalized to GAPDH (n = 3). (**G**) Drug retention was decreased in B16-F10 cells pretreated with bGH for 1 week (n = 3). (**H**) Similar results were found in SK-MEL-30 cells (n = 3). Data are presented as mean ± standard deviation (*, *p* < 0.05, Mann-Whitney U test). (**I**) Representative images for SK-MEL-30 cells with fluorescent dye (green) treated with and without 50 ng/mL hGH for 1 week (20× magnification; scale bar represents 300 μm). (**J**) Relative RNA levels for seven different ABC efflux pumps in B16-F10 tumors grown in vivo in bGH and WT mice, normalized against reference genes (both sexes combined). bGH mice (n = 12), WT mice (n = 12). (**K**) Relative RNA levels for seven different ABC efflux pumps in B16-F10 tumors grown in vivo in GHRKO and WT mice, normalized against reference genes (both sexes combined). GHRKO mice (n = 10), WT mice (n = 13). (**L**) Heatmap showing the variations in RNA expression of ABC efflux pumps in tumors in bGH or GHRKO mice (both sexes combined). Data are presented as mean ± standard error (*, *p* < 0.05, unpaired student’s *t*-test).

We analyzed the effect of GH action on the expression levels of ABC transporter expression both in cell culture as well as in the tumor lysates from male and female bGH and GHRKO vs. their corresponding controls. The RNA expression levels of different ABC-type pumps based on their known critical role in conferring chemoresistance [58,64,65,66,67,68,69,70] were quantified. In B16-F10 cultured cells, the RNA levels of *Abcb1a*, *Abcg1* and *Abcg2* were markedly upregulated by 5-fold, 3-fold and 2-fold respectively, following 24- and 48-h exposure to 50 or 500 ng/mL bGH compared to untreated controls (Figure 3A–C). In human SK-MEL-30 melanoma cells treated with 250 ng/mL hGH in vitro increased expression of *ABCB5*, *ABCB8*, *ABCG1 and ABCG2* RNA (Figure 3D). At the protein level, B16-F10 cells treated with bGH showed an upregulation of the expression of ABCB1, ABCG1, and ABCG2 (Figure 3E and Figure S9), similar to the trend in RNA expression. We also observed a robust increase at the protein level of ABCB1 and ABCG2 in the SK-MEL-30 cells in a dose-dependent manner with GH (Figure 3F and Figure S9). In vitro, we also assessed IGF-1 dose needed for elevated RNA expression of multiple ABC transporters. IGF-1 only modestly and non-significantly increased mainly the *Abcc* type transporter levels (Figure S4E and Table 1). To test whether the GH induced increase in ABC transporter levels lead to a biological effect of increased efflux capacity, we performed a drug retention assay using DiOC2 dye as a drug surrogate. The results indicate ABCB1 and to a lesser extent ABCC1 and ABCG2 transporter activities. Both B16-F10 and SK-MEL-30 cells, showed 12–15% lower (Figure 3G) and 12–19% lower (Figure 3H,I) dye retained respectively in GH treated samples compared to controls (Figure 3G–I).

In vivo, we found that both ABC transporter RNA and protein expression were elevated in the implanted melanoma tumors in bGH and GHRKO mice relative to controls. The RNA levels of ABC transporters in tumors from bGH mice were upregulated significantly for *Abcb8* (*p* = 0.041), *Abcc2* (*p* = 0.011), *Abcc4* (*p* = 0.021), *Abcg1* (*p* = 0.047), and *Abcg2* (*p* = 0.016) (Figure 3J,L). In addition, the tumors from GHRKO mice had significantly higher levels of *Abcb1a* (*p* = 0.008), *Abcb8* (*p* = 0.020), *Abcg1* (*p* = 0.033), and *Abcg2* (*p* = 0.021) (Figure 3K,L), relative to their WT littermates. As summarized in the heatmap (Figure 3L), tumors from bGH mice, with high GH and high IGF-1, had a general increase in multiple ABC transporters tested. Whereas in the tumors from GHRKO mice, with high GH but very low IGF-1, the increase was lost specifically for *Abcc2* and *Abcc4*, suggesting that: (i) IGF-1, might be particularly responsible for the increase in *Abcc2* and *Abcc4* expression in tumors in vivo; and (ii) GH itself, independent of IGF-1, can increase the other ABC transporters including *Abcb1a*, *Abcb8*, *Abcg1* and *Abcg2*, consistent with the in vitro results (Figure 3A–I). Further analyses revealed a few sex-specific differences in GH/IGF-induced expression of ABC transporters in bGH and GHRKO mice (Figure S5). 

In male bGH mice, *Abcb1a* (*p* = 0.038) and *Abcg1* (*p* = 0.0001) were significantly upregulated (Figure S5A), while in the female bGH mice with significantly higher tumoral IGF-1 production (Figure 2I), five ABC transporters (*Abcb8*, *Abcc1*, *Abcc2*, *Abcc4*, and *Abcg2*; *p* = 0.004, 0.015, 0.002, 0.015, and 0.009, respectively) were significantly upregulated (Figure S5B). Interestingly, in the GHRKO mice, the males had significantly higher *Abcb1a* (*p* = 0.0001), *Abcb8* (*p* = 0.017), *Abcg1* (*p* = 0.016), and *Abcg2* (*p* = 0.015) (Figure S5C), while the females with elevated tumoral GH (Figure 2H) had significantly higher *Abcg2* (*p* = 0.035) but none of the *Abcc*-type transporters (Figure S5C,D). At the protein level, the ABCB1 expression was confirmed to be significantly upregulated in tumors from male GHRKO mice, indicating a GH specific effect (*p* = 0.025) (Figures S5F and S9). We previously reported that GH-mediated upregulation of ABC-type multidrug efflux pumps is particularly enhanced when the cells are treated with chemotherapeutics like doxorubicin [58]. Here, even at the basal stage (i.e., prior to any drug-exposure), the tumors in bGH and GHRKO mice had markedly higher RNA expression of different groups of ABC-type drug efflux pumps when compared to their WT counterparts (Figure 3L), indicating that GH specifically increases expression of the *Abcb* and *Abcg* groups of drug efflux pumps, while IGF-1 increases expression of the *Abcc* group of drug efflux pumps. We suggest that both GH and IGF-1 can selectively prime the tumors to be drug resistant.

In this regard, we reviewed and compiled all of the published studies on GH, IGF-1, and both of their effects on ABC transporter expression in different cancers (Table 1). Notably, unlike the current study, none of the earlier studies compared GH vs. IGF-1 effects. Cross comparison of the reported effects of GH and IGF-1 on ABC transporter expression in different cancers (including results from this study) are shown in Table 1 and indicates that IGF-1 has an impact in particularly elevating the *Abcc* group of ABC transporters, while GH elevates the *Abcb* and *Abcg* groups.

### 2.4. Elevated GH Drives Expression of Epithelial-to-Mesenchymal Transition (EMT) Markers In Vitro and In Vivo

**Figure 4:**
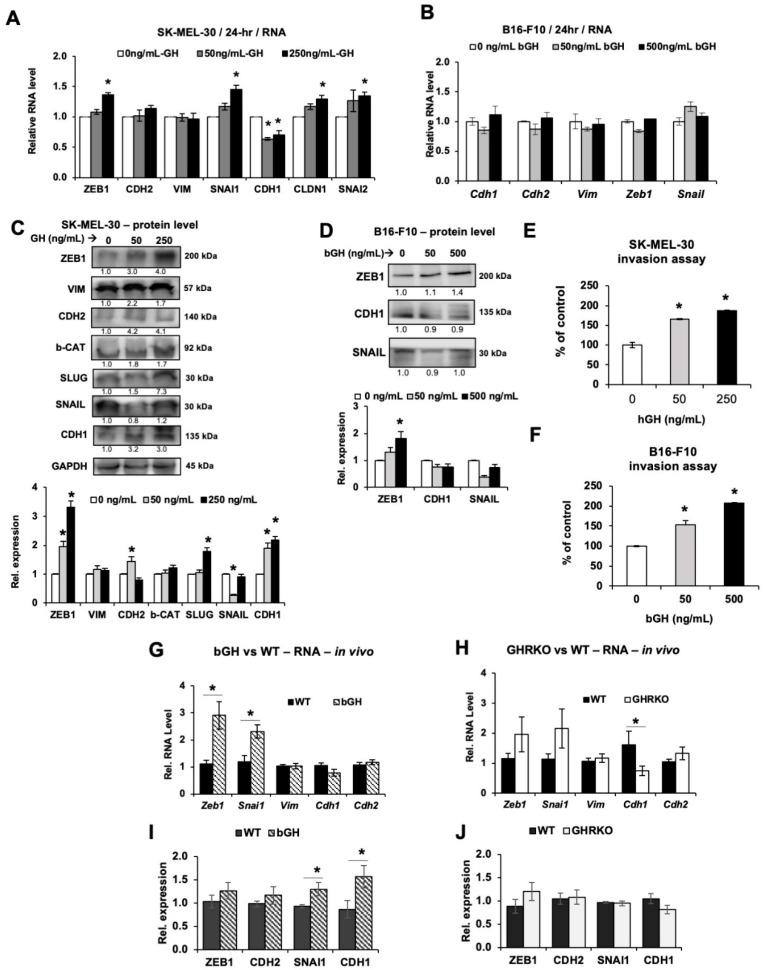
Changes in markers of epithelial-to-mesenchymal transition (EMT) in melanoma in vitro and in vivo. (**A**) Changes in RNA levels in SK-MEL-30 cells treated with hGH for 24 h, normalized against *GAPDH* gene. (**B**) Relative RNA levels in B16-F10 cells treated with bGH for 24 h, normalized against reference genes. (**C**) Proteins levels of EMT markers in SK-MEL-30 cells after hGH treatment for 48 h, normalized to GAPDH. (**D**) Proteins levels of EMT markers in B16-F10 cells after bGH treatment for 72 h, normalized against total protein. (**E**,**F**) Invasion of SK-MEL-30 (**E**) and B16-F10 (**F**) cells after 7-days hGH or bGH pretreatment. Fold changes are presented and refer to control. Data are presented as mean ± standard deviation (n = 3; *, *p* < 0.05, Mann-Whitney U test). (**G**,**H**) Relative RNA levels of EMT markers in B16-F10 tumors grown in vivo in bGH (**G**) or GHRKO (**H**) mice, normalized against reference genes (both sexes combined). (**I,J**) Proteins levels of EMT markers in tumors from WT, bGH (**I**) or GHRKO (**J**) mice. Representative images are shown in Figure S6F,G. (both sexes combined; bGH mice n = 12, WT mice n = 12; GHRKO mice n = 10, WT mice n = 13). Data are presented as mean ± standard error (*, *p* < 0.05, unpaired student’s t-test).

We analyzed the effect of GH action on the expression levels of EMT markers and transcription factors expression both in cell culture as well as in the tumor lysates from bGH vs. WT and GHRKO vs. WT male and female mice. We previously showed that in human melanoma cells in vitro exogenously added GH upregulates markers of EMT while attenuating GHR expression decreases the EMT induction [57]. Here we queried known markers of EMT in both human and mouse melanoma cells treated with GH. After 24-h GH treatment, SK-MEL-30 human melanoma cells showed a modest but significant increase of multiple EMT-related genes including mesenchymal markers *ZEB-1*, *SNAI1*, *CLDN1* and *SNAI2*, while the epithelial marker *CDH1* was significantly downregulated (Figure 4A). At the protein level, the mesenchymal markers ZEB-1, CDH2 and SNAI2 were all markedly higher following GH treatment in SK-MEL-30 (Figure 4C and Figure S9). In B16-F10 mouse melanoma cells, although a 24-h bGH treatment did not significantly increase the EMT markers (Figure 4B), at the protein level, we saw a significant increase in ZEB-1 (Figure 4D and Figure S9). Since GH does not induce the expression of IGF-1 in cultured B16-F10 cells in vitro (Figure 1A and Figure S1E), the regulation of ZEB-1 expression in cultured cells is possibly a direct effect of GH, independent of IGF-1. An increase in mesenchymal markers indicates an increase in the invasion capacity of the tumor cells. Using a basement membrane invasion assays, we saw that both B16-F10 cells and SK-MEL-30 cells did have a GH dose dependent increase in invasion capacity compared with control cells (Figure 4E,F), which corroborates the known effect of GH in promoting the process of EMT in normal and cancer tissues [76].

An assessment of the GH and IGF-1 effects on EMT in vivo, revealed significant overlap between GH and IGF-1 in driving EMT (Figure 4G,H). In the B16-F10 tumors derived from bGH mice, RNA expression analyses showed significant upregulation of the mesenchymal transcription factors *Zeb1* (*p* = 0.020) and *Snai1* (*p* = 0.035) (Figure 4G), but only similar, non-statistically significant trends in the GHRKO mice (Figure 4H). However, the epithelial marker *Cdh1* was significantly suppressed in the GHRKO vs. the WT mice. Several previous studies have reported IGF-1 to be a potent inducer of the EMT process [60]. As shown in the heatmap (Figure S6H), at the RNA level, the trend of EMT marker changes in tumors from bGH and GHRKO mice was similar, implying that GH can directly induce EMT, independent of IGF-1. In vitro IGF-1 treatment alone markedly elevated the RNA levels of *Zeb1* (Figure S6E). Sex-specific RNA analyses further showed a marked increase of *Zeb1* (*p* = 0.008) and *Snai1* (*p* = 0.010) in male bGH mice and of *Zeb1* (*p* = 0.028) in the female bGH mice, while male GHRKO mice had elevated *Snai1* compared to the WT controls (Figure S6A–D). We also observed a significant increase in SNAI1 and CDH1 protein levels in bGH mice when compared to controls (Figure 4I), but not in GHRKO mice (Figure 4J). In Table 2, we summarized all previous studies and work presented here on the effects of GH or IGF-1 in EMT induction in various types of cancer in vitro or in vivo. As is readily observable in Table 2, both GH and IGF-1 are potent inducers of EMT, with significant overlap in suppressing epithelial markers and upregulating several mesenchymal factors.

## 3. Discussion

GHR expression is elevated in a wide range of cancers [8,17,18,19,21], especially in the case of melanoma [56], implicating the potential involvement of GH in this process. Previously, we had reported that not only do melanoma cells overexpress GHR but also that targeting GHR in human melanoma cells attenuates tumor progression, EMT induction, drug efflux and more importantly, sensitizes these cells to chemotherapy in vitro [57,58]. Additionally, enhanced metastasis of mouse melanoma cells to the lungs in response to locally elevated GH levels in DJ-1 knockout mice was recently reported and confirms a role of GHR in melanoma metastases [4]. Our current study provides a first in vivo validation to our earlier in vitro mechanistic observations on the role of GH in melanoma chemoresistance.

Locally produced GH and IGF-1 in the tumor microenvironment imparts autocrine and paracrine effects to drive tumor growth in multiple ways [49]. A series of studies by Lobie et al. have highlighted that autocrine GH is potent in mediating GH action comparative to endocrine GH, in breast cancer [11,47,48,51,52,53,54,77]. Due to the complexity of the endocrine features of the GH/IGF-1 axis, it is difficult to distinguish between the direct effects of GH and its indirect effects via IGF-1. Mouse B16-F10 cells are compatible with the C57BL/6J mice with high-GH-high-IGF-1 (bGH mice) and high-GH-low-IGF-1 (GHRKO mice) in vivo, and allowed a distinctive opportunity to tease out specific effects of GH or IGF-1 [54].

Recently, we reported that knocking down the GHR attenuated both ABC-type multidrug efflux pump gene expression and markers of EMT in multiple human melanoma cell lines, thereby improving drug efficacy [57,58]. In melanoma, the activation of the EMT strongly correlates with not only a transition to aggressive metastases [60,93], but also with an upregulation of mechanisms of drug resistance [58,94]. Our observations in this report, using a syngeneic mouse model of melanoma, corroborated existing reports of GH/IGF effects in human melanoma cells (Table 1 and Table 2). EMT appeared to be driven by both GH and IGF-1 (Figure 4 and Table 2). However, we observed that GH or IGF-1 independently affect different groups of ABC-type multidrug efflux pump expression in our mouse models (Figure 3 and Table 1). A significant upregulation of markers of EMT mediators as well as ABC drug efflux pumps, even in the absence of any drug mediated induction in this study, suggests a critical role of GH in establishing an intrinsic therapy resistance in melanoma. The RNA levels of *Abcb1a*, *Abcg1* and *Abcg2*, some of the most studied drug transporters in cancer, were elevated in bGH and GHRKO mice, both of which have elevated circulating GH irrespective of the levels of IGF-1. Besides the endocrine GH and IGF-1 levels, the tumoral production of GH and IGF-1 in vivo as depicted in Figure 2G–J is of importance. Together, the results strongly suggest that GH has a direct effect in upregulating *Abcb1a*, *Abcb8*, *Abcg1*, and *Abcg2* expression independent of IGF-1 in vivo (Table 1). The intense drug resistance which is the hallmark feature of aggressive forms of melanoma could thus be a function of GH action. We also identified that IGF-1 appears to particularly upregulate the *Abcc* subtype transporters in contrast to a direct GH effect (Figure 3 and Table 1). Indeed, previous studies showed that suppression of IGF-1R decreases ABCC2 expression and promoted drug sensitivity in human colon cancer cells [74], and that IGF-1 increased the expression of ABCC1, ABCC2, and ABCC3 in both leukemia cells and ovarian cancer cells [73,75]. This study is the first to compare the different effects of GH and IGF-1 in melanoma in vivo. It is important to note that GH has IGF-1-independent effects in upregulating subgroups of ABC transporters (Table 1). Recently a GH induced chemoresistance by upregulation of ABCG2 has been recently validated in breast cancer as well [71].

Recently, Caramel et al. [95] had shown that a switch from a Zeb2-dominant phenotype to an EMT-inducing Zeb1-dominant phenotype is a driver of malignancy in melanoma. Furthermore, Zeb1 has also been identified as a critical oncogenic regulator in uveal melanoma [96]. It has been shown that downregulation of IGF-1 decreased levels of ZEB1 and CDH2/N-cadherin associated with increased levels of CDH1/E-cadherin and MITF, the major regulator of melanocyte differentiation [60]. In our study, we confirmed a marked and consistent increase in levels of GH-induced *Zeb1* in vivo, under both conditions of elevated GH (in bGH and GHRKO mice) as summarized in Table 2. These data along with our observations of elevated *Snai1/Snail* and reduced *Cdh1/E-cadherin* in this syngeneic mouse model additionally point to a role of GH in driving lineage plasticity or phenotypic switch in cancer cells [76]. As shown in Table 2, GH and IGF-1 have been separately shown to induce EMT in various types of cancers both in vitro and in vivo, including breast, lung, colon, and glioblastoma. Our results suggest that both GH and IGF-1 can induce EMT, while IGF-1 is perhaps more potent.

An interesting finding of our study were sex-specific differences between the patterns of altered expression of ABC-type multidrug efflux pumps and EMT markers in male and female mice with elevated GH—a critical area of focus in the development of more personalized therapies. The role of estrogen, differences in the pattern of GH release between male and female mice, and a putative role of a differential IGF axis could be potential confounding factors in choice of therapy. Also, GH exerts several sexually dimorphic gene expressions in several tissues [97,98].

Despite changes in tumor gene expression and increased cell growth in response to GH treatment in vitro, we did not observe increased tumor growth or size due to excess GH or IGF-1 in vivo. There could be several possible reasons for this observation. First, the endogenous GH/IGF levels in WT mice could be adequate to drive tumor growth and may mask the effects of supraphysiological levels of GH or IGF-1. Additionally, tumoral production of GH and IGF-1 poses another compounding factor in this problem. This could be addressed in the future with smaller tumor inoculum in the mice. However, in relevance to our observations of melanoma tumor growth in the mouse models of high-GH-high-IGF-1 (bGH mice) and high-GH-low-IGF-1 (GHRKO mice), we further analyzed melanoma patient data from the human TCGA database using the GEPIA platform [99]. To assess the effects of GH responsivity in determining survival among melanoma patients with high and low GHR expression, we compared survival between melanoma patients within the top and bottom quartiles for GHR expression level. We observed no significant differences in either overall or disease-free survivals in the Kaplan-Meier plots between these two patient groups. To assess effects of IGF-1 responsivity in determining patient survival, we conducted a similar comparison between melanoma patients within the top and bottom quartiles for IGF-1R expression levels and obtained similar results. This indicates that neither GHR nor IGF-1R expression appears to be a determinant of overall mortality in melanoma patients in the TCGA cohort (Figure S7), though they may influence treatment response and outcome, as the expression of several EMT mediators and ABC transporters were upregulated in the TCGA dataset irrespective of the heterogeneity in treatment patterns (Figure S8). A controlled study with combination of drug doses and GHR antagonists or GH treatment in vivo could validate the theory. Lastly, in this regard it is worthwhile to refer to a hallmark report from Green et al. in 1985, where they presented a dual-effector theory of GH action [100]—stating that GH drives cellular differentiation while IGF-1 drives the clonal expansion. Thus, homeostatic interactions between GH and IGF-1 and subsequent net effects in cancer is unexplored despite being very provocative towards explaining oncogenic processes like EMT, drug efflux, apoptosis, cancer stemness, and metastases where GH and IGF-1 overlap.

Two independent laboratories have reported that congenital GH insensitivity, as found in patients with Laron Syndrome (LS), imparts a dramatic resistance to cancer in humans [101,102]. This is corroborated by the high degree of cancer resistance in GHRKO, or “Laron mice”, and other GH deficient dwarf mouse models [1,14,16]. Additionally, scores of in vitro, in vivo, epidemiological and clinical studies have validated the ‘oncodriver’ role of GH-GHR interaction in several human cancers [39,103]. Within the normal population, the potential anti-cancer effects of suppressing the GH-axis are still underappreciated. Numerous scientific studies have now shown that GH drives progression of breast and endometrial [3,9,11,15,24,25,46,47,48,49,50,51,52,53,54], colorectal [18,19], thyroid [22], and hepatocellular carcinoma [23]. GHR inhibition, using the GHR antagonist, Pegvisomant, alone has been found to attenuate the growth of both colon and breast cancers [10,104]. Additionally, recent research has strongly implicated GH action in driving cancer therapy resistance [40]. It is important to note that recombinant hGH replacement therapy in GH deficient patients with no prior report of cancer, has been found safe, and is not associated with any significant increases in cancer risk [6,105,106,107,108,109,110]. On the other hand, the role of IGF-1 in stimulating tumor progression is well established, as numerous studies have shown that elevated, and even basal IGF-1 levels are strongly associated with detrimental cancer prognosis and therapy resistance [105,106,111,112,113,114]. Decreased IGF-1 levels reduce rates of malignancy [115,116] and the inhibition of IGF-1R inhibits cancer growth [60,117]. Accordingly, several IGF-1R inhibitors have reached different stages of anti-cancer clinical development; yet none have been approved for clinical application due to complications like hyperglycemia and hyperinsulinemia [118]. Therefore, GHR inhibition appears to be a safer and validated point of intervention to suppress cancer growth and especially drug resistance, as well as in reducing circulating IGF-1 levels and improving insulin sensitivity [63,119,120,121]. Here, we confirmed that both GH and IGF-1 have independent as well as overlapping actions in the intrinsic therapy refractoriness of melanoma. To our knowledge this is the first in vivo report of the tumor-promoting mechanisms of GH and IGF-1 in melanoma.

## 4. Materials and Methods

### 4.1. Cell Culture and GH

B16-F10 mouse melanoma cells (CRL-6475, ATCC, Manassas, VA, USA) and mouse L cell fibroblast (CRL-2648, ATCC) were purchased from ATCC. SK-MEL-30 human melanoma cells (CSC-C0302, Creative Bioarray, Shirley, NY, USA) were purchased from Creative Bioarray. The cells were maintained in high glucose DMEM or EMEM, with 10% FBS (complete growth media) and 1× penicillin-streptomycin. Cells were passaged twice a week (passage numbers are <20). Recombinant bovine GH (#CYT-636, ProspecBio, East Brunswick, NJ, USA) was used in B16-F10 cells; recombinant human GH (#ABIN9344914, Antibodies Online, Limerick, PA, USA) was used in SK-MEL-30 cells. Following preliminary GH dose-response studies spanning several orders of magnitude (5, 50, and 500 ng/mL), the 500 ng/mL dose of bGH elicited the greatest response and was thus selected for further experiments using the B16-F10 mouse cell line. The doses of hGH used in SK-MEL-30 cells were 50 and 250 ng/mL. For short-term GH signaling studies, B16-F10 or SK-MEL-30 cells were seeded on 6-well plates and incubated overnight in complete growth media. On the second day, the media was replaced with serum-free media for 4 h prior to the treatment of bGH or hGH as indicated. In longer-term treatment (24, 48 or 72 h), cells were incubated with bGH or hGH in 2% FBS media, as previously described [57,58,59].

### 4.2. Real-Time RT-qPCR

Total RNA from cells and tumor tissues was isolated (GeneJET RNA Purification kit, #K0732 Thermo Fisher Scientific, Waltham, MA, USA), quantified using the BioAnalyzer 2100 (Agilent, Santa Clara, CA, USA), and reverse transcribed to cDNA (Thermo Fisher Scientific Maxima Enzyme Mix, #K1642, 5× Reaction Mix, #R1362) as previously described [122]. Maxima SYBR Green/Fluorescein qPCR Master Mix (Thermo Fisher Scientific, #K0243) was employed to quantitatively measure the abundances of target RNA in the samples using an iCycler (BioRad, Hercules, CA, USA). Qbase was used for data analysis. The net RNA levels of *Gh*, *Ghr*, *Igf1* and *Igf-1r* are presented in 2^(-dCt) format. Relative RNA levels are presented in 2^(-ddCt) format and normalized against reference genes (*Gapdh*, *B2m, Eif3f* or *Hprt*). Fold changes are shown in relative to controls. The primer sequences are listed in Table S1.

### 4.3. Immunocytochemistry

Cells were seeded at 10,000 cells/cm^2^ in 8-well chamber slides and fixed with 4% freshly-prepared formaldehyde (pH 6.9) for 15 min at room temperature (RT) as previously done [58]. After fixation, the cells were permeabilized with 0.2% Triton-X100 in phosphate buffered saline (PBS) for 15 min at RT, followed by blocking with 1% BSA, glycine and 0.1% Tween-20 in PBS for 2 h at RT. Then cells were incubated with primary antibody at 1:250 dilution at 4 °C for 12 h, washed four times with 0.1% Tween-20 containing PBS (PBST) and incubated with secondary antibody (1:1000 dilution) at RT for 1 h. Finally, the slides were washed four times with PBST and the samples were mounted with DAPI-containing Fluoroshield mounting medium (#ab104139, Abcam, Cambridge, UK). Rabbit anti-human GHR monoclonal antibody (Abcam #ab134078) and goat anti-rabbit secondary antibody with AlexaFluor488 tag (#R37116, Life Technologies, Carlsbad, CA, USA) were used. Microscopic imaging was done using Cytation 3 imaging reader (BioTek, Winooski, VT, USA) and Gen5 2.09 software. The scale bar represents 100 μm. Magnification was at 20×.

### 4.4. Knockdown of GHR Expression by siRNA

B16-F10 mouse melanoma cells were transfected with small interfering (si) RNA (GHR siRNA: #4390771, #4390771, Thermo Fisher Scientific; #NM_010284, Sigma Aldrich, St. Louis, MO, USA) targeting the GHR gene using Lipofectamine RNAi MAX Transfection Reagent (Thermo Fisher Scientific, #13778075). Control cells were transfected with scrambled control siRNA (Thermo Fisher Scientific, Scramble siRNA: #4390843). Efficiency of *GHR* RNA knockdown was verified by real-time RT-qPCR and confirmed via western blotting for GH-induced tyrosine phosphorylated STAT5 (CST #9351). Following the knockdown of GHR in the cell line, proliferation assays were performed to test the effect of GH on cell growth. The GHR of human SK-MEL-30 cells were knocked down similarly using pre-designed GHR-targeting siRNA from Origene (Rockville, MD, USA) and verified by real-time RT-qPCR. A universal scrambled siRNA duplex (Origene #SR30004) was used as control.

### 4.5. Western Blotting

Protein extracted from B16-F10 and SK-MEL-30 cells treated with or without GH, and tumors was tested for the phosphorylation of STAT5, ERK1/2, AKT, SRC, CDH1, CDH2, ZEB1, SNAI2, SNAIL/SNAI1, VIM, b-CAT, t-ERK1/2, t-AKT, ABCC1, ABCB1, ABCG2, IGF1R beta, p-STAT1, t-STAT1, p-STAT3, t-STAT3, t-STAT5 (CST #9351, #4370, #4058, #2101, #3195, #13116, #3396, #9585, #3879, #2101, #5741, #8480, #9102, #4685, #72202, #13342, #42078, #3027, #9167, #14994, #9145, #12640, #94205), and ABCG1 (NB400-132, Novus Biologicals, Littleton, CO, USA) to determine their activation or expression induced by GH as previously described [123]. β-Actin, GAPDH (#4970, #5174, CST, Danvers, MA, USA) or total protein staining (#926-11011, LI-COR, Lincoln, NE, USA) were used as loading controls. Densitometry analysis of individual blots was performed using Image Studio LITE Ver 5.2 (LI-COR, Lincoln, NE, USA). The relative expression levels (fold change relative to controls) are labeled under each band of representative images.

### 4.6. Cell Proliferation Assay

Approximately ten thousand B16-F10 or SK-MEL-30 cells were seeded on 96-well plates and incubated overnight in complete growth media. On the second day, the media was replaced with serum-free media for 4 h. Cells were incubated for an additional 48 h in 2% FBS media with various doses of bGH or hGH. Cell proliferation was determined using a Resazurin Cell Viability Assay (Sigma Aldrich) [124]. For cell proliferation assays in the presence of siRNA, approximately 5000 cells were seeded per well and transfected with siRNA in complete growth media. After 48 h, the cells were washed and treated with 500 ng/mL bGH or 50 and 250 ng/mL hGH in 2% FBS for 48 h. At the end of this assay, cell viability was determined.

### 4.7. Cellular Metabolism

Extracellular acidification rate (ECAR) and oxygen consumption rate (OCR) of the cells or tumor tissues were measured using a Seahorse XFe24 Analyzer (Agilent). The cells were seeded in 24-well plates overnight and incubated with serum-free media for 4 h followed by a 24-h incubation in serum-free media containing bGH. The ECAR and OCR of the cells were then analyzed using a Glycolysis Stress Test and Mito Stress Test (Agilent) as previously described [125]. Tumor tissues collected at dissection were sliced and weighed before their basal levels of ECAR and OCR were measured in Seahorse assay media (102365-100, Agilent). The measurements were normalized to the weight of each sample.

### 4.8. Mouse Models of Subcutaneous Melanoma

12-week-old male and female GHRKO and bGH mice, as well as their respective WT controls, all in a C57BL/6J genetic background were used [37,38,126]. These syngeneic mice are widely used for the evaluation of B16-F10 melanoma cells injected subcutaneously in vivo [127]. In this study, 5 million cells were subcutaneously inoculated into the flank of each mouse. The GHRKO and bGH mice were housed with their WT littermate controls. The lengths of perpendicular tumor diameters were measured every other day using a digital caliper as previously described [126]. Tumor volume was calculated using the formula: tumor size = 0.5 × (length) × (breadth)^2^ [126]. Male bGH (n = 5), WT mice (n = 6); female bGH (n = 7), WT mice (n = 6); male GHRKO (n = 6), WT (n = 8); female GHRKO (n = 4), WT (n = 5). After the mice were sacrificed, tumors were surgically removed, weighed, frozen in liquid nitrogen and stored at −80 °C until RNA and protein was extracted and analyzed. Animal studies were performed in accordance to policies of the Ohio University Institutional Animal Care and Use Committee and fully complied with all federal, state and local policies(16-H-016).

### 4.9. Enzyme-Linked Immunosorbent Assay (ELISA)

To detect released IGF-1 levels in supernatants from cultured cells, B16-F10 cells were seeded on 12-well plates and incubated overnight in complete growth media. On the second day, the media was removed, and cells were rinsed with PBS twice. Then cells were incubated with various doses of bGH in serum-free media for 24 or 48 h. Afterward, supernatants of cultured cells were collected and measured by ELISA for IGF-1 (#22-IG1MS-E01, ALPCO, Salem, NH, USA). For tumor GH and IGF-1 measurements, lysed tumor proteins were collected and quantified by Bradford assay. GH and IGF-1 were quantified by ELISA kits (#22-GHOMS-E01; #22-IG1MS-E01, ALPCO) and normalized to total protein concentrations.

### 4.10. Drug Retention Assay

B16-F10 or SK-MEL-30 cells were pretreated with GH in 2% FBS-containing DMEM or EMEM for a week, the media being freshly prepared and replaced every other day. On the assay day, cells were trypsinized, counted and suspended at 1 million cells per mL in cold DiOC_2_(3) dye on ice for 15 min (#ECM910, Chemicon International, Thermo Fisher Scientific). The cells were then centrifuged, and the supernatant removed. The cell pellets were then subsequently resuspended in cold efflux buffer and distributed into different Eppendorf tubes under the following conditions: one set of tubes was kept on ice to deactivate the drug-efflux pumps as controls, while the other two sets were kept in a 37 °C water bath for 30 min and 120 min, respectively, allowing the active drug-efflux pumps to drive out the DiOC_2_(3) dye. Afterward, the cells were centrifuged and washed. Cell suspensions were dispensed into the wells of a black-wall-clear-bottom 96-well plate and measured in a fluorescence plate reader at an excitation wavelength of 485 nm and an emission wavelength of 530 nm.

### 4.11. Invasion Assay

B16-F10 or SK-MEL-30 cells were pretreated with GH in 2% FBS-containing DMEM or EMEM for one week. The media was freshly prepared and changed every other day. On the day of the invasion assay, the cells were trypsinized and counted. One hundred thousand cells were then seeded per well in the upper chamber of the CytoSelect 96-well Cell Invasion Assay kit (CBA-112, Cell Biolabs, Inc., San Diego, CA, USA) and incubated with GH in serum-free media for 24 h. The cells from the underside of the membranes were then dislodged, lysed and stained with CyQuant GR dye solution. The fluorescence for each well at 480/520 nm, which correlates with cell number, was determined using a fluorescence plate reader.

### 4.12. Statistics

All in vitro experiments were repeated at least thrice. For in vitro experiments, we performed normality test, test of homogeneity of variance, followed by the Mann-Whitney U test using the RStudio v4.0.1 software (RStudio, Boston, MA, USA). For in vivo experiments, we performed normality test, test of homogeneity of variance, followed by the unpaired student’s t-test. Tumor sizes were analyzed by repeated measures (SPSS Statistics 17.0, Chicago, IL, USA). Significance was set as a *p* value ≤ 0.05.

## 5. Conclusions

**Figure 5:**
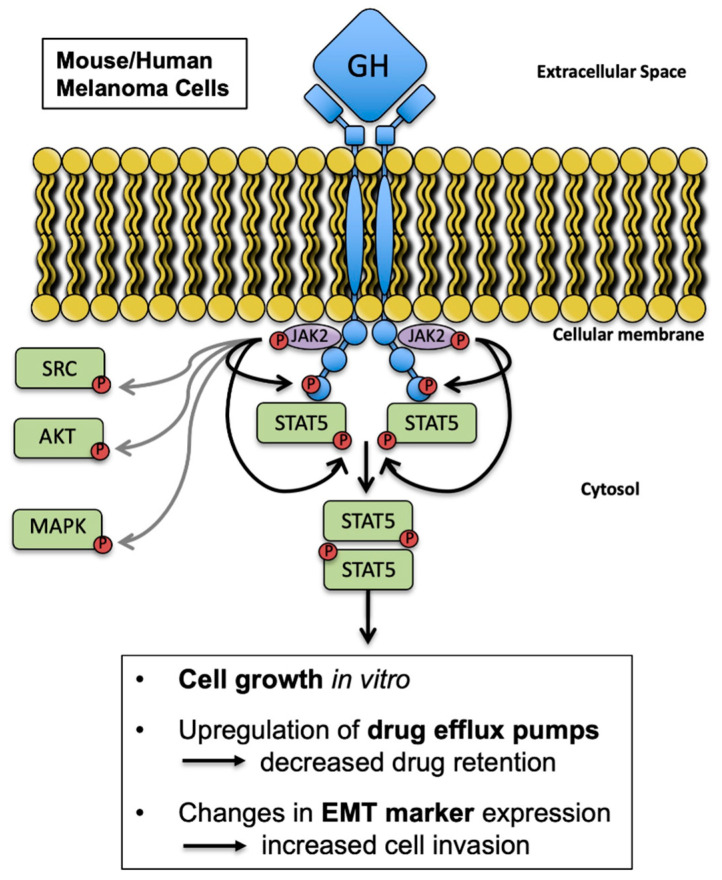
Proposed model for this study. GH stimulates melanoma growth in vitro through GHR activation independent of IGF-1. In the syngeneic mouse melanoma models, elevated GH, independent of IGF-1, alters tumor expression of multidrug efflux pumps and epithelial-mesenchymal transition markers in vivo.

In summary, the current study utilized unique syngeneic murine melanoma models of altered GH/IGF expression and represents the first study of the effects of GH on melanoma in vivo. While elevated GH did not accelerate growth of implanted melanoma tumors in vivo, cellular analyses showed that elevated GH activates oncogenic signaling, upregulates the intrinsic capacity of the tumor for drug clearance and metastatic features (Figure 5). The current study is the first to suggest that IGF-1 has a role in specifically elevating the Abcc group of ABC transporters, while GH specifically elevates Abcb and Abcg type ABC-transporters in melanoma in vivo. Also, both GH and IGF-1 are potent inducers of EMT with significant overlap in suppressing multiple epithelial markers and upregulating several mesenchymal factors. We believe that this study strengthens the foundations of GH-driven drug resistance and warrants future studies in combining GHR antagonism with chemotherapy in treating melanoma.

## Figures and Tables

**Table 1:** Summary of known effects of GH/IGF-1 axis on ABC transporters in different cancers.

**Effects of GH**	**Observed Effects**	**Refs**
**Cancer Type**		
Breast Cancer (In vivo)	Increased ABCG2	[71]
Human Melanoma (In vitro)	Increased ABCB1, ABCB5, ABCB8, ABCC1, ABCC2, ABCG1, ABCG2	[58] Current study
Mouse Melanoma (In vitro)	Increased *Abcb1a*, *Abcg1*, *Abcg2*	Current study
Mouse Melanoma (In vivo)	Increased *Abcb1a*, *Abcb8*, *Abcg1*, *Abcg2*	Current study
**Effects of IGF-1**	**Observed Effects**	**Refs**
**Cancer Type**		
Ovarian cancer (In vitro and vivo)	Increased ABCB1, ABCC1, ABCC2, ABCC3, ABCC5, ABCG2	[72,73]
Colorectal cancer (In vitro)	Increased ABCC2	[74]
Leukemia (In vitro)	Increased ABCB1, ABCC1, ABCC2, ABCC3, ABCG2	[75]
Mouse Melanoma (In vivo)	Increased *Abcc2*, *Abcc4*	Current study

Italicized gene names represent RNA in mouse; capitalized gene names represent human RNA or proteins.

**Table 2:** Summary of the effects of GH and IGF-1 on EMT mediators in different cancers.

**Effects of GH**	**Observed Effects**	**Refs**
**Cancer Type**		
Breast Cancer (In vitro and in vivo)	Increased SNAIL, SLUG, VIM, CDH2, FN1 Decreased CDH1, OCLN, CTNNA1, JUP	[77,78]
Endometrial adenocarcinoma (In vitro)	Increased FN1 Decreased CTNNA1	[24]
Colorectal cancer (In vitro and in vivo)	Increased SNAIL, TWIST2, FN1 Decreased CDH1, OCLN	[45,79]
Pancreatic ductal adenocarcinoma (In vitro)	Increased SNAIL, SLUG, VIM, ZEB1, CDH2, CTNNB2 Decreased CDH1	[21]
Human Melanoma (In vitro)	Increased SNAIL, SLUG, VIM, ZEB1, CDH2, CLDN1 Decreased CDH1	[57] Current study
Mouse Melanoma (In vivo)	Increased *Zeb1*, *Snai1* Decreased *Cdh1*	Current study
**Effects of IGF-1**	**Observed Effects**	**Refs**
**Cancer Type**		
Breast Cancer (In vitro and in vivo)	Increased SNAIL, VIM, ZEB1, CDH2, TWIST1, FN1 Decreased CDH1, OCLN	[80,81]
Colorectal cancer (In vitro and in vivo)	Increased SNAIL, VIM, CDH2 Decreased CDH1, ZO-1	[82,83]
Lung cancer (In vitro)	Increased SNAIL, VIM, CDH2, FN1, CTNNB1 Decreased CDH1	[84,85]
Gastric cancer (In vitro)	Increased SNAIL, ZEB2, CDH2	[86,87,88]
Prostate cancer (In vitro)	Increased ZEB1, VIM, CDH2, FN1, CTNNB1 Decreased CDH1	[89,90]
Liver cancer (In vitro)	Increased SNAIL, VIM, CDH2 Decreased CDH1	[91]
Glioblastoma (In vitro)	Increased VIM, CDH2 Decreased CDH1, ZO-1	[92]
Human Melanoma (In vitro)	Increased ZEB1, CDH2 Decreased CDH1	[60]
Mouse Melanoma (In vitro)	Increased *Zeb1*	Current study
Mouse Melanoma (In vivo)	Increased *Snai1*, *Zeb1* Decreased *Cdh1* (m)	Current study

Italicized gene names represent RNA in mouse; capitalized gene names represent human RNA or proteins. (m) indicates males only.

## Supplementary Materials

The following are available online at https://www.mdpi.com/2072-6694/12/12/3640/s1. Figure S1: Melanoma cells are responsive to GH treatment in vitro. Figure S2: Relative RNA expression of *Ghr*, *Igf-1*, and *Igf-1r* in B16-F10 mouse melanoma tumors from bGH and GHRKO mice. Figure S3: GH does not alter the metabolism of either mouse melanoma cells treated with GH for 24 hours or mouse melanoma tumors in bGH and GHRKO mice. Figure S4: Genotypic changes in ABC efflux pump expression in B16-F10 mouse melanoma in vitro. Figure S5: Genotypic changes in ABC efflux pump expression in B16-F10 mouse melanoma tumors in vivo. Figure S6: Genotypic changes in markers of epithelial-to-mesenchymal transition (EMT) in B16-F10 mouse melanoma in vivo. Figure S7: Kaplan-Meier plots for overall and disease-free survivals in human melanoma patients (TCGA dataset). Figure S8: Levels of fold-change in EMT mediators and ABC transporters in human melanoma patients (TCGA dataset). Figure S9: Original images for Western blotting. Table S1: Primer sequences for real-time RT-qPCR and siRNA sequences for mouse and human genes. Table S2: Tumor weight and organs from bGH transgenic and WT C57BL/6J mice at dissection. Table S3: Tumor weight and organs from GHRKO and WT C57BL/6J mice at dissection.
