# Supplementary material for: Growth Hormone Upregulates Mediators of Melanoma Drug Efflux and Epithelial-to-Mesenchymal Transition In Vitro and In Vivo
Source: Cancers (Basel). 2020 Dec 4;12(12):3640. doi: 10.3390/cancers12123640 (PMC7761932; doi:10.3390/cancers12123640)
Supplement: Supplementary file 1 [file cancers-12-03640-s001.pdf]

Supplementary files

Growth Hormone Upregulates Mediators of Melanoma Drug Efflux and Epithelial-to-Mesenchymal Transition In Vitro and In Vivo

Yanrong Qian [1,†], Reetobrata Basu [1,†], Samuel C. Mathes [1,2], Nathan A. Arnett [1,3], Silvana Duran-Ortiz [1,2,4], Kevin R. Funk [1,2,4], Alison L. Brittain [1,4,5], Prateek Kulkarni [1,2,4], Joseph C. Terry [1,2], Emily Davis [1,2,4], Jordyn T. Singerman [1,2], Brooke E. Henry [1], Edward O. List [1], Darlene E. Berryman [1,5] and John J. Kopchick [1,4,5,*]

Figure S1

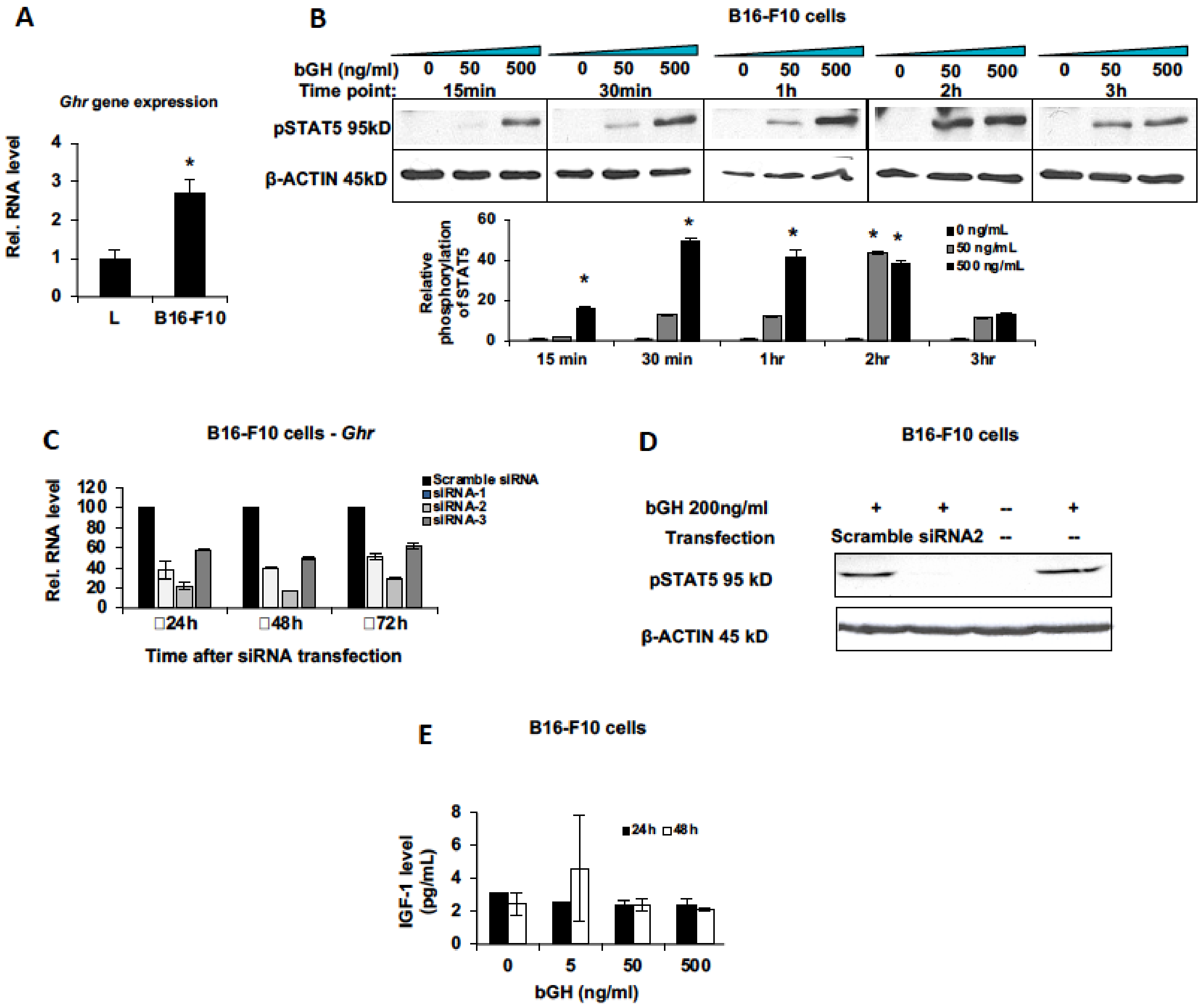

Figure S1. Melanoma cells are responsive to GH treatment in vitro. (A). Relative RNA expression of growth hormone receptor (*Ghr*) in B16-F10 mouse melanoma cells compared with L cell mouse fibroblast cells by real-time RT-qPCR. The net RNA levels of *Ghr* is presented in 2^(-dCt) format (n=3). **(B).** Phosphorylated STAT5 in B16-F10 mouse melanoma cells treated with bGH for short term (15 minutes to 3 hrs). B16-F10 cells were treated with bGH for different lengths of time as indicated. Densitometry analysis of individual blots was performed using Image Studio LITE Ver 5.2. Protein levels were normalized against the expression β-Actin. Quantification are shown in bar graphs (n=3). **(C).** Efficiency of GHR knock down by siRNA. In order to knockdown the GHR, three different GHR-specific siRNAs were tested, and the efficiency of each was evaluated at various time points relative

to a non-specific siRNA (Scramble siRNA) by using realtime RT-qPCR to measure the relative expression levels of *Ghr* mRNA. Relative RNA levels are presented in 2^(-ddCt) format. RNA expression was normalized against reference genes. (n=3). **(D)**. Down regulation of phosphorylated STAT5 by knock down of GHR (n=3). **(E).** Released IGF-1 in serum-starved B16-F10 cells treated with bGH measured by ELISA (n=3). Data are presented as mean ± standard deviation (*, p<0.05, Mann-Whitney U test).

Figure S2

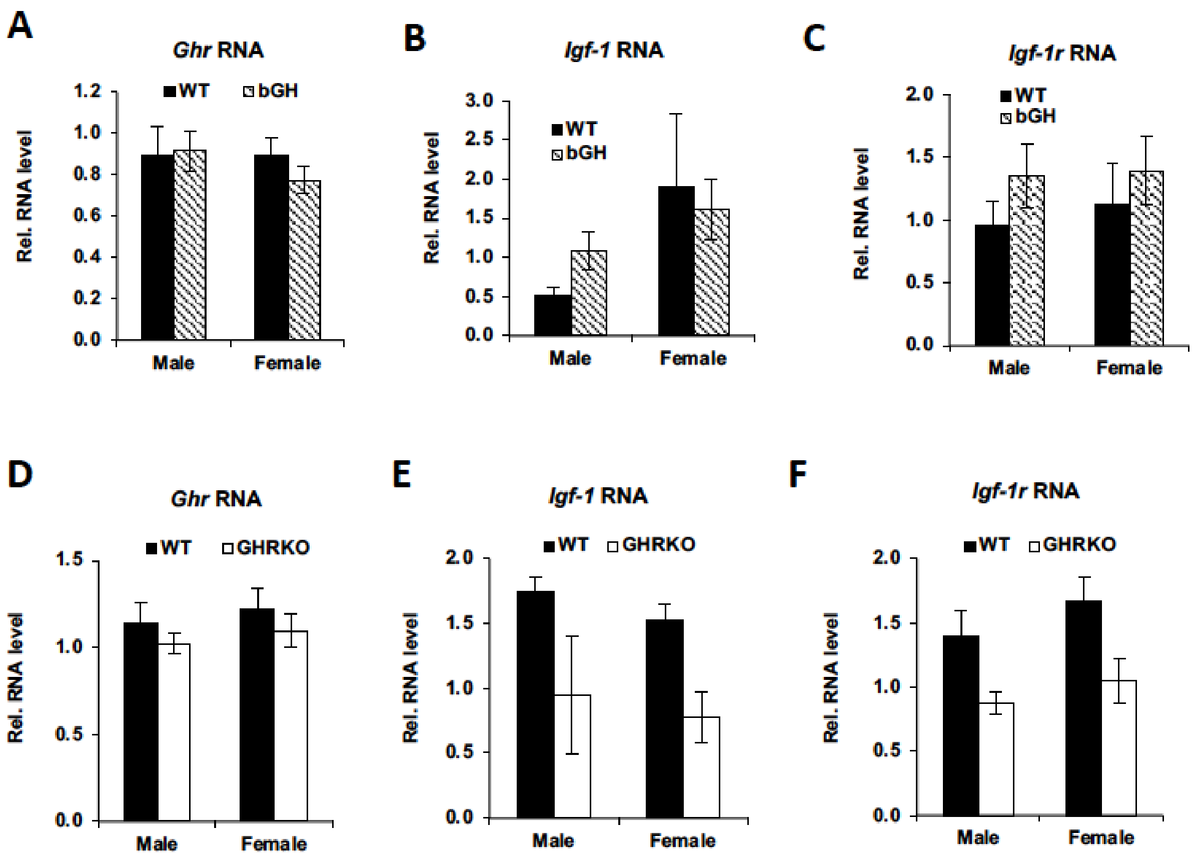

Figure S2. Relative RNA expression of *Ghr, Igf-1, and Igf-1r* in B16-F10 mouse melanoma tumors from bGH and GHRKO mice. (A). Relative expression of *Ghr* RNA in tumors from bGH mice. Male bGH (n=5) mice vs male WT mice (n=6). Female bGH (n=7) and female WT mice (n=6). (**B).** Relative expression of *Igf-1* RNA in tumors from bGH mice. Male bGH (n=5) mice vs male WT mice (n=6). Female bGH (n=7) and female WT mice (n=6). (**C).** Relative expression of *Igf-1r* RNA in tumors from bGH mice. Male bGH (n=5) mice vs male WT mice (n=6). Female bGH (n=7) and female WT mice (n=6). (**D)**. *Ghr* RNA in tumors from GHRKO mice. **(E).** Expression of *Igf-1* RNA in tumors from GHRKO mice. **(F).** Expression of *Igf-1r* RNA in GHRKO mice. Male GHRKO mice (n=6) vs male WT mice (n=8); Female GHRKO mice (n=4) and female WT mice (n=5). Relative RNA levels are presented in 2^(-ddCt) format. RNA expression was normalized against reference genes. Data are presented as mean ± standard error (unpaired student's t-test).

Figure S3

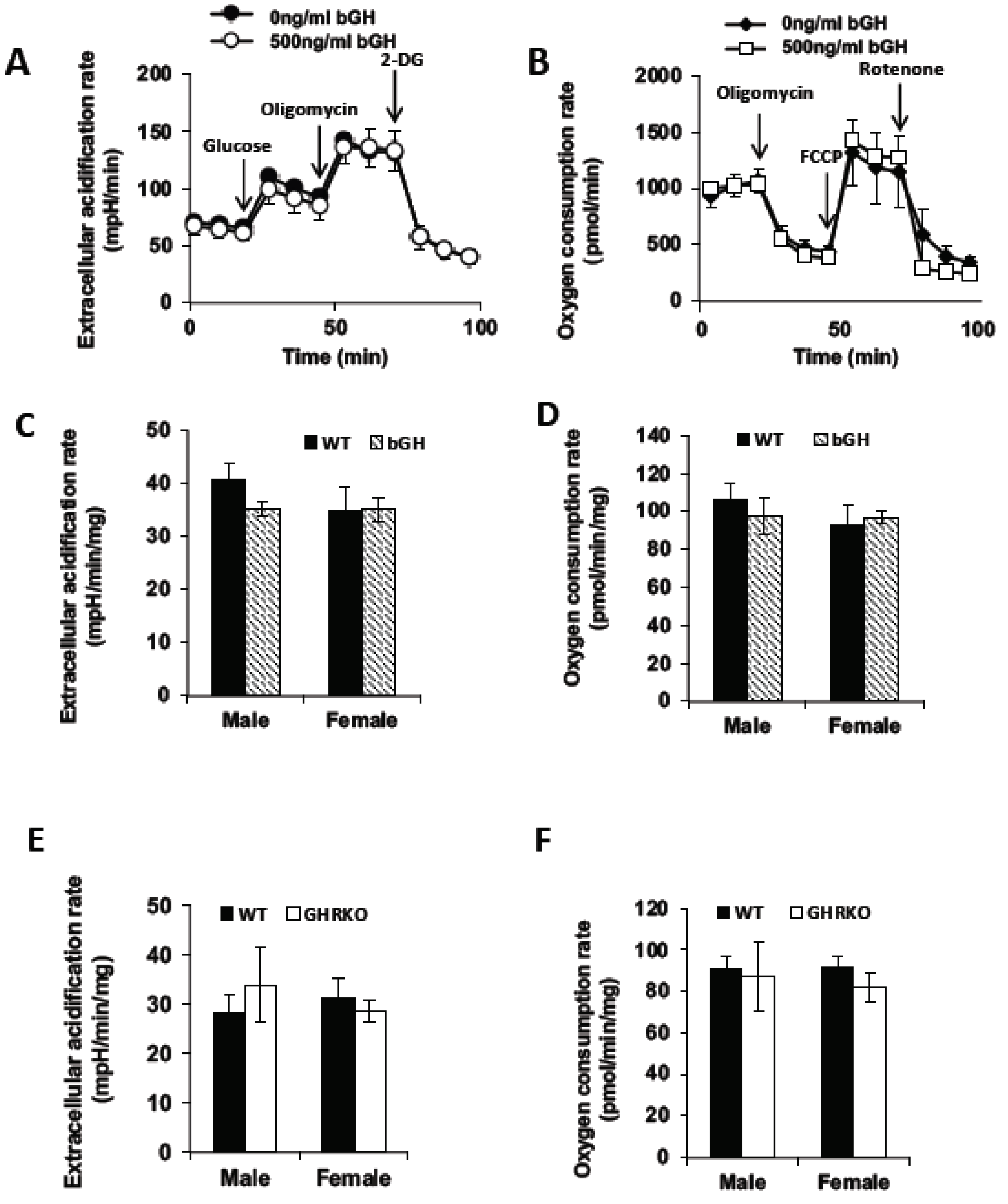

Figure S3. GH does not alter the metabolism of either mouse melanoma cells treated with GH for 24 hours or mouse melanoma tumors in bGH and GHRKO mice. (A). The glycolysis rate of cultured B16-F10 cells was not affected by bGH treatment for 24 hours. Data are presented as mean ± standard deviation (n=3-4; Mann-Whitney U test). (B). Oxidative phosphorylation of cultured B16-F10 cells was not affected by GH treatment for 24 hours. Data are presented as mean ± standard deviation (n=3-4). (C-D) ECAR and OCR of melanoma slices from bGH and WT mice (n=4-6). (E-F) ECAR and OCR of melanoma slices from GHRKO and WT mice (n=4-6). The measurements were normalized to tumor weight per sample. Data are presented as mean ± standard error (unpaired student's t-test).

Figure S4

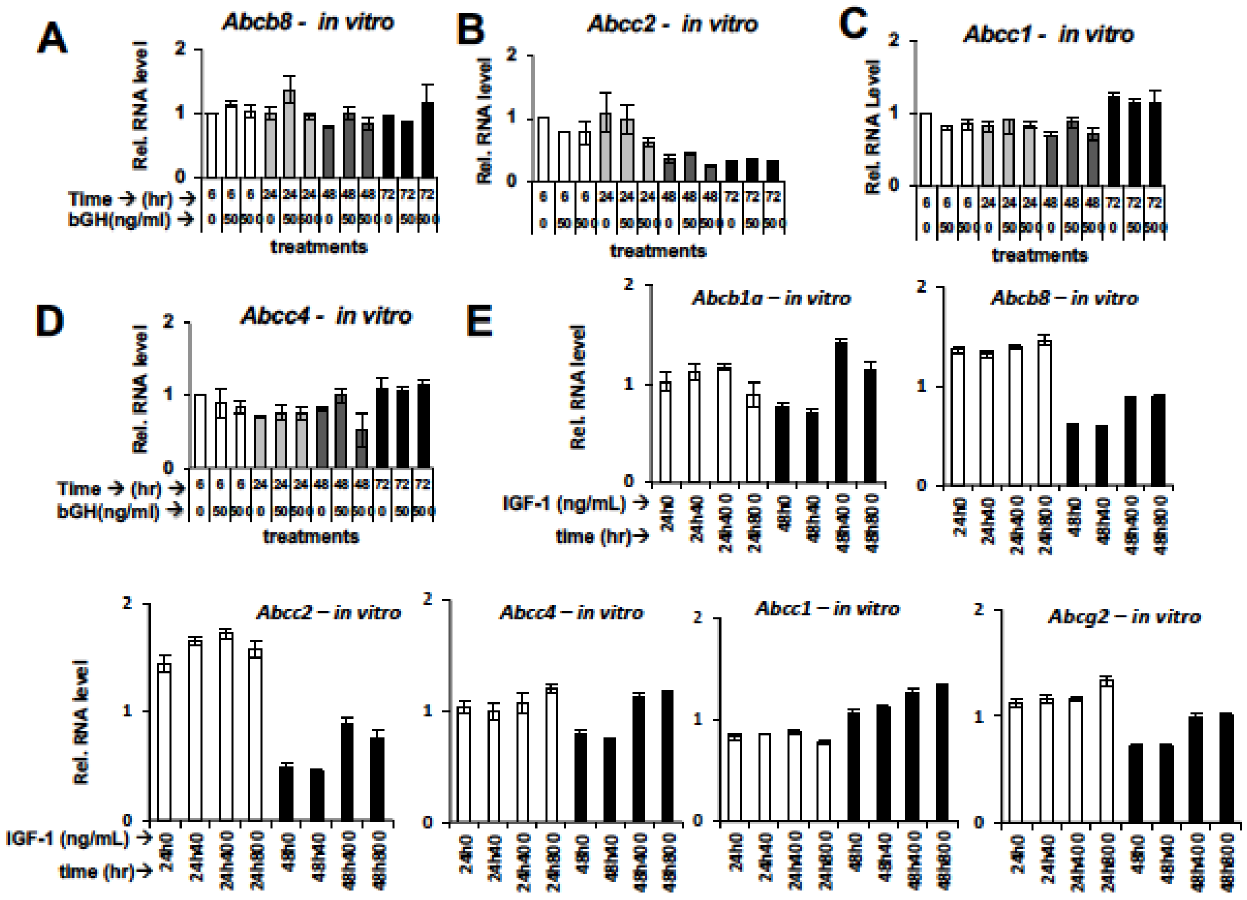

Figure 4. Genotypic changes in ABC efflux pump expression in B16-F10 mouse melanoma in vitro. (A-D). Relative RNA levels in B16-F10 mouse melanoma cells treated with 0, 50 or 500 ng/mL bGH for 6, 24, 48 and 72 hours, were queried by real-time RT-qPCR for different ABC efflux pumps. Relative RNA levels are presented in 2^(-ddCt) format. RNA expression was normalized against reference genes (n=3). **(E).** Relative RNA levels in B16-F10 mouse melanoma cells treated with 0, 40, 400, or 800 ng/mL IGF-1 for 24 and 48 hours, were queried by real time RT-qPCR. Relative RNA levels are presented in 2^(-ddCt) format. RNA expression was normalized against reference genes (n=3). Data are presented as mean ± standard deviation (Mann-Whitney U test).

Figure S5

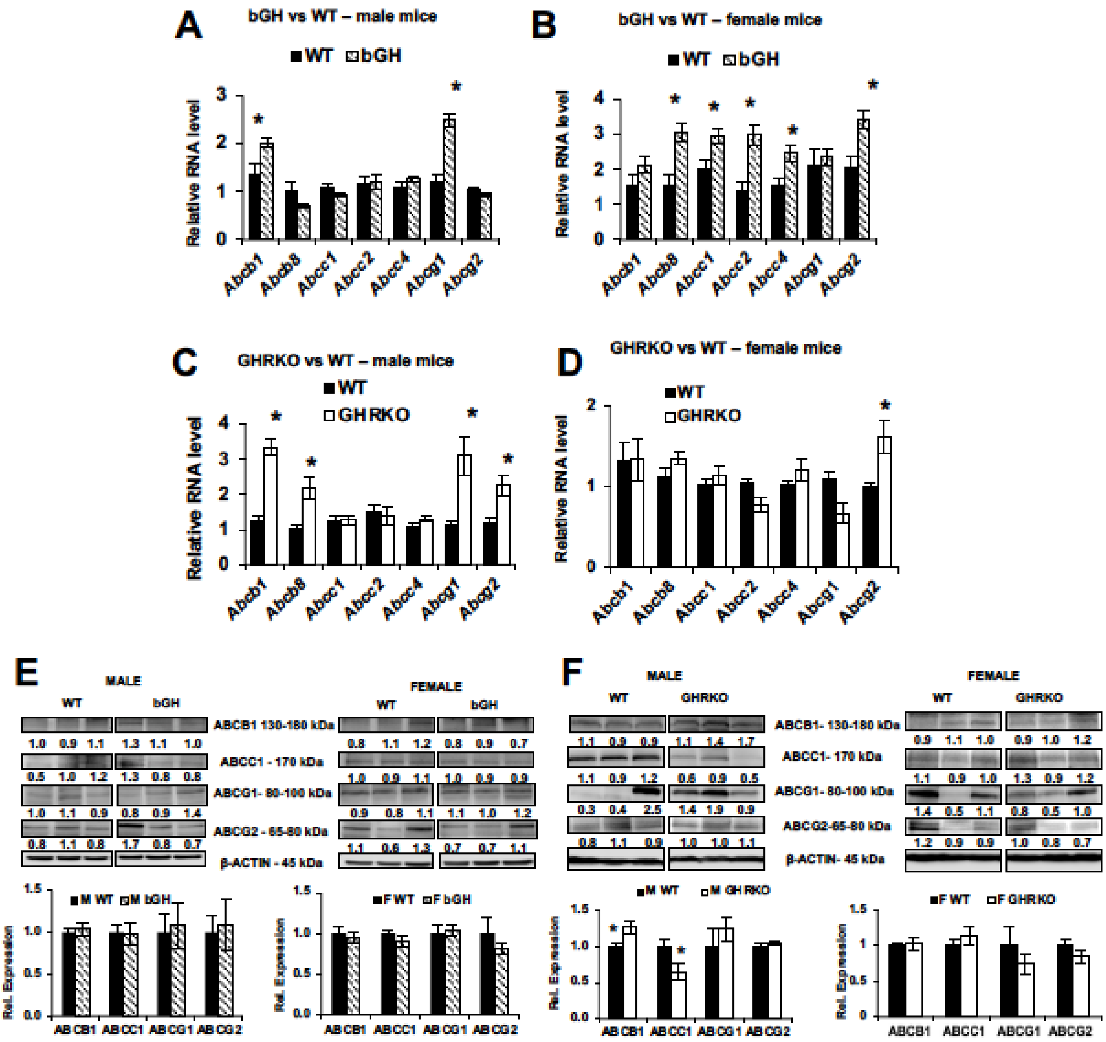

G

protein expression – *in vivo*

protein	bGH		GHRKO	
ABCB1	1.0		1.2	*
ABCB8	–		–	
ABCC1	1.0		0.9	
ABCC2	–		–	
ABCC4	–		–	
ABCG1	1.0		1.1	
ABCG2	1.0		1.0	

fold-change
2.0
1.5
1.0
0.8
0.5

Figure S5. Genotypic changes in ABC efflux pump expression in B16-F10 mouse melanoma tumors in vivo. Relative RNA levels in B16-F10 mouse melanoma tumors grown *in vivo* in bGH male **(A)**, bGH female **(B)**, GHRKO male **(C)**, and GHRKO female **(D)** mice were queried by real-time RT-qPCR for seven different ABC efflux pumps. Relative RNA levels are presented in 2^(-ddCt) format. RNA expression was normalized against reference genes. bGH male (n=5), WT male (n=6), bGH female (n=7), WT female (n=6); GHRKO male (n=6), WT male (n=8), GHRKO female (n=4), WT female (n=5). **(E-F).** Proteins levels of ABC efflux pumps of tumors from WT and bGH mice or GHRKO mice. WB was performed using appropriate antibodies. Densitometry analysis of individual blots was performed using Image Studio LITE Ver 5.2. Protein levels were normalized against the expression β-Actin. The relative expression levels (fold change) are labeled under each band. Quantification are shown in bar graphs. bGH male (n=5), WT male (n=6), bGH female (n=7), WT female (n=6); GHRKO male (n=6), WT male (n=8), GHRKO female (n=4), WT female (n=5). **(G)** Heatmap showing the variations in protein expression of ABC efflux pumps in tumors in bGH or GHRKO mice (both sexes combined). bGH mice (n=12), WT mice (n=12). GHRKO mice (n=10), WT mice (n=13). Data are presented as mean ± standard error. (* as compared with WT mice, p≤0.05, unpaired student's t-test).

Figure S6

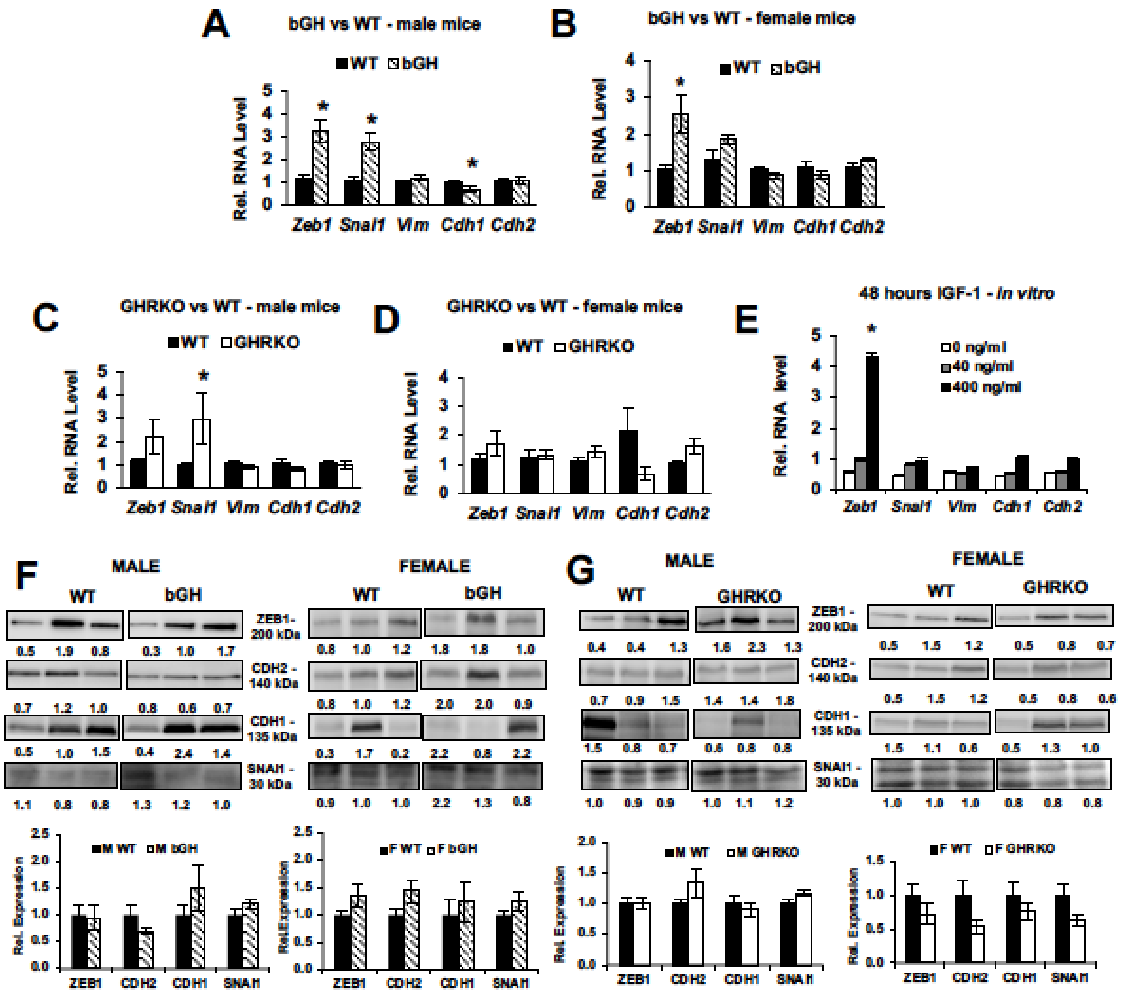

H

RNA	bGH	GHRKO
Zeb1	2.6	1.7
Snai1	1.9	1.9
Vim	1.0	1.1
Cdh1	0.7	0.5
Cdh2	1.1	1.3

0.8	1.0	1.3

Fold change

Protein	bGH	GHRKO
ZEB1	1.2	0.9
SNAI1	1.3	1.0
VIM	n.d.	n.d.
CDH1	1.3	0.9
CDH2	1.0	0.8

0.8	1.0	1.2

Fold change

Figure S6. Genotypic changes in markers of epithelial-to-mesenchymal transition (EMT) in B16-F10 mouse melanoma in vivo. Relative RNA levels in B16-F10 mouse melanoma tumors grown *in vivo* in bGH male (A), bGH female (B), GHRKO male (C), and GHRKO female (D) mice were queried by real-time RT-qPCR for five known markers of EMT. Relative RNA levels are presented in 2^(-ddCt) format. RNA expression was normalized against reference genes. bGH male (n=5), WT male (n=6), bGH female (n=7), WT female (n=6); GHRKO male (n=6), WT male (n=8), GHRKO female (n=4), WT female (n=5). Data are presented as mean ± standard error (*, p<0.05, unpaired student's t-test). (E). Relative RNA levels in B16-F10 mouse melanoma cells treated with 0, 40, or 400 ng/mL IGF-1 for 48 hours were queried by real-time RT-qPCR. Relative RNA levels are presented in 2^(-ddCt) format. RNA expression was normalized against reference genes (n=3). Data are presented as mean ± standard deviation. (* as compared with control; *, p<0.05, Mann-Whitney U test). (F-G) Proteins levels of EMT markers of tumors from WT, bGH (F) or GHRKO (G) mice. WB was performed using appropriate antibodies. Densitometry analysis of individual blots was performed using Image Studio LITE Ver 5.2. Protein levels were normalized against total protein. The relative expression levels (fold change) are labeled under each band. Quantification are shown in bar graphs. bGH male (n=5), WT male (n=6),

bGH female (n=7), WT female (n=6); GHRKO male (n=6), WT male (n=8), GHRKO female (n=4), WT female (n=5). Data are presented as mean ± standard error. (H). Heat-map showing the variations in RNA and protein expression of EMT markers in tumors in bGH or GHRKO mice (both sexes combined; unpaired student's t-test).

Figure S7

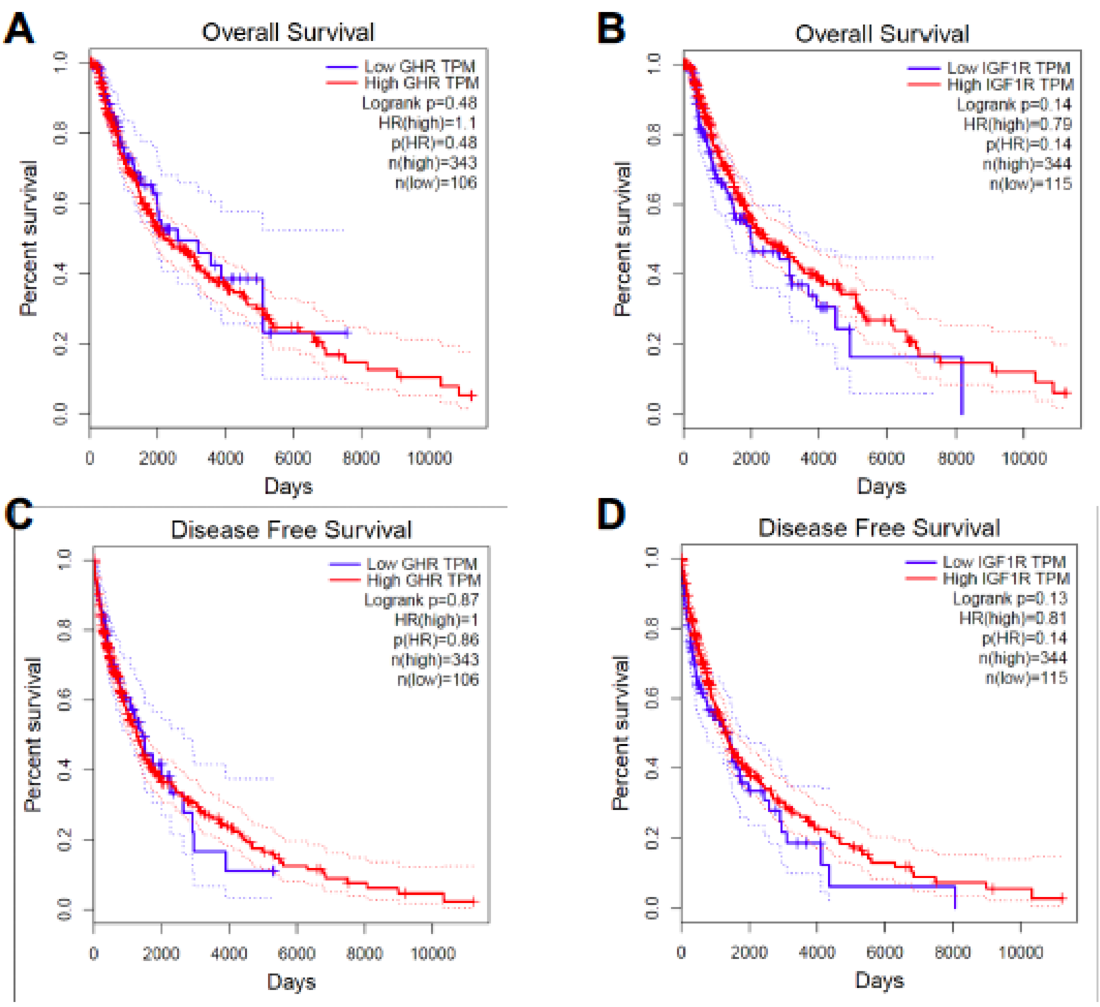

Figure S7: Kaplan-Meier plots for overall and disease-free survivals in human melanoma patients (TCGA dataset). Using the GEPIA platform, KM-plots were generated for human melanoma patients in the TCGA dataset to compare overall and disease-free survival between melanoma patients at the top-25% and bottom-25% of either GHR **(A,C)** or IGF1R **(B,D)** expression levels.

Figure S8

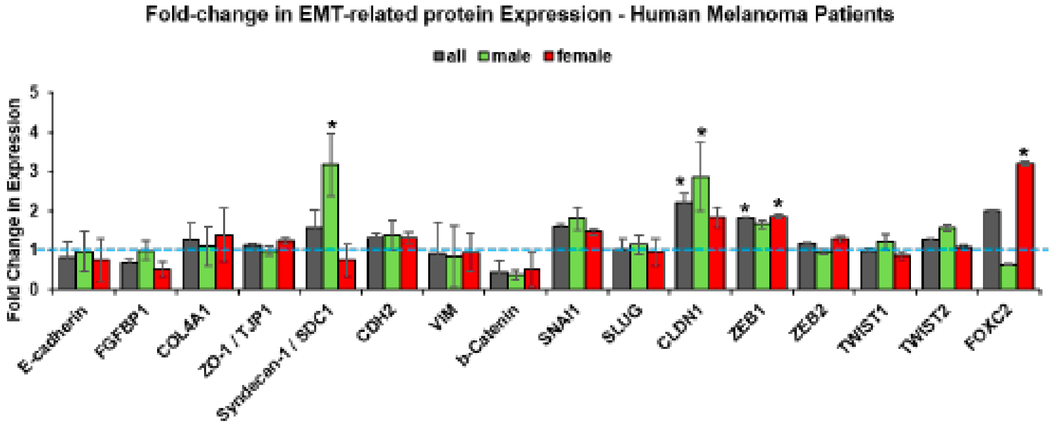

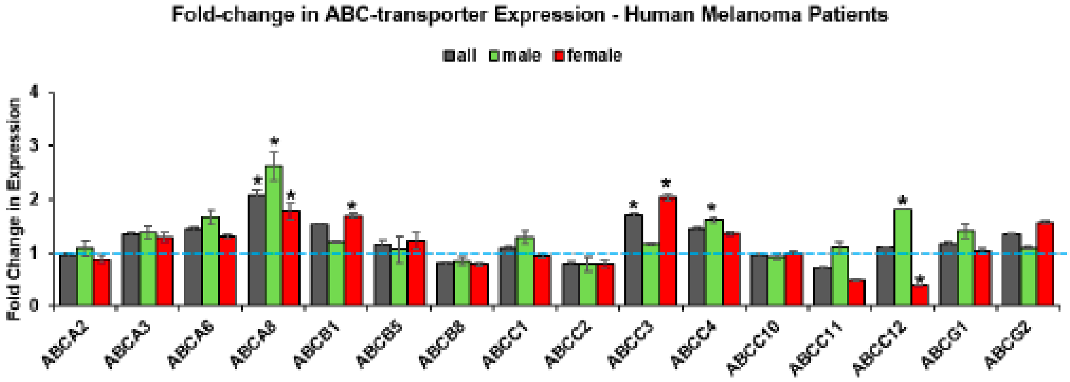

Figure S8: Levels of fold-change in EMT mediators and ABC transporters in human melanoma patients (TCGA dataset).

Fig. S9. Original images for Western blotting.

Original images for Fig. 1D-F

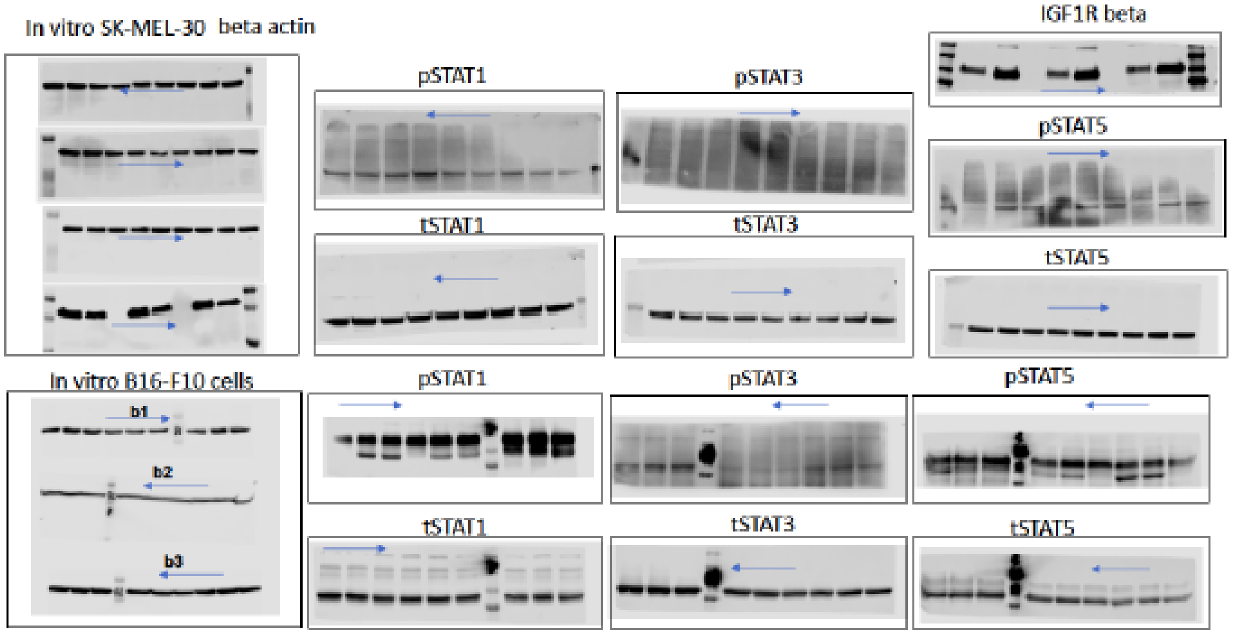

Notes: Numbers indicate different gels or blots. Arrow indicates the orientation of sample loading.

Original images for Fig. 1G, 1H, S1B, S1D

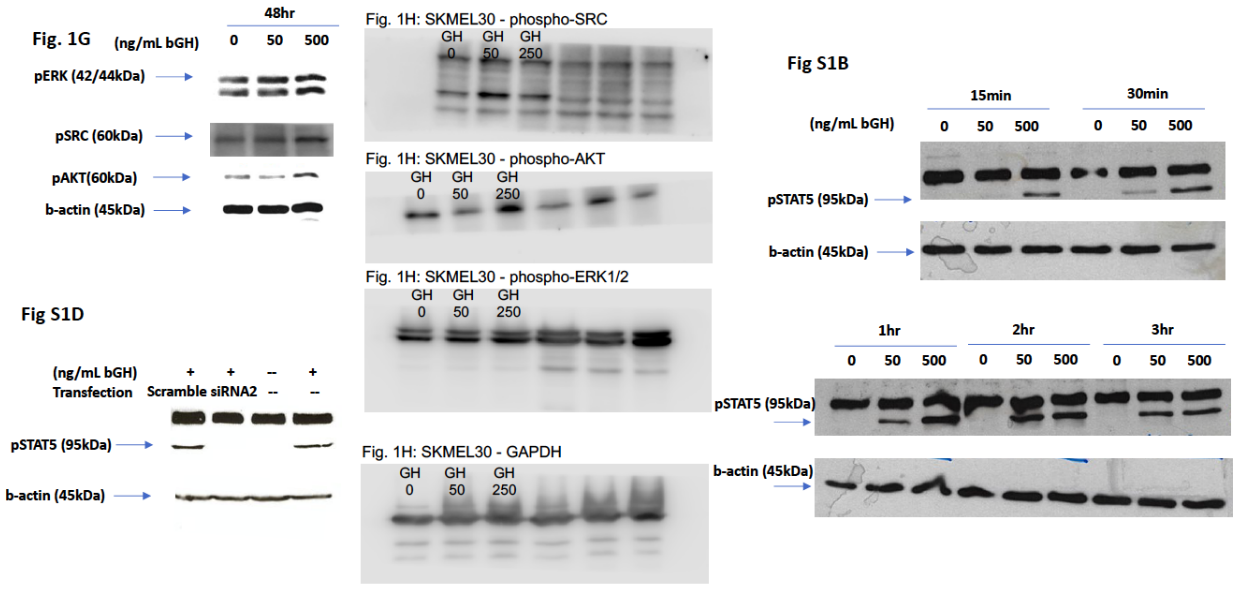

Fig. S9. Original images for Western blotting.

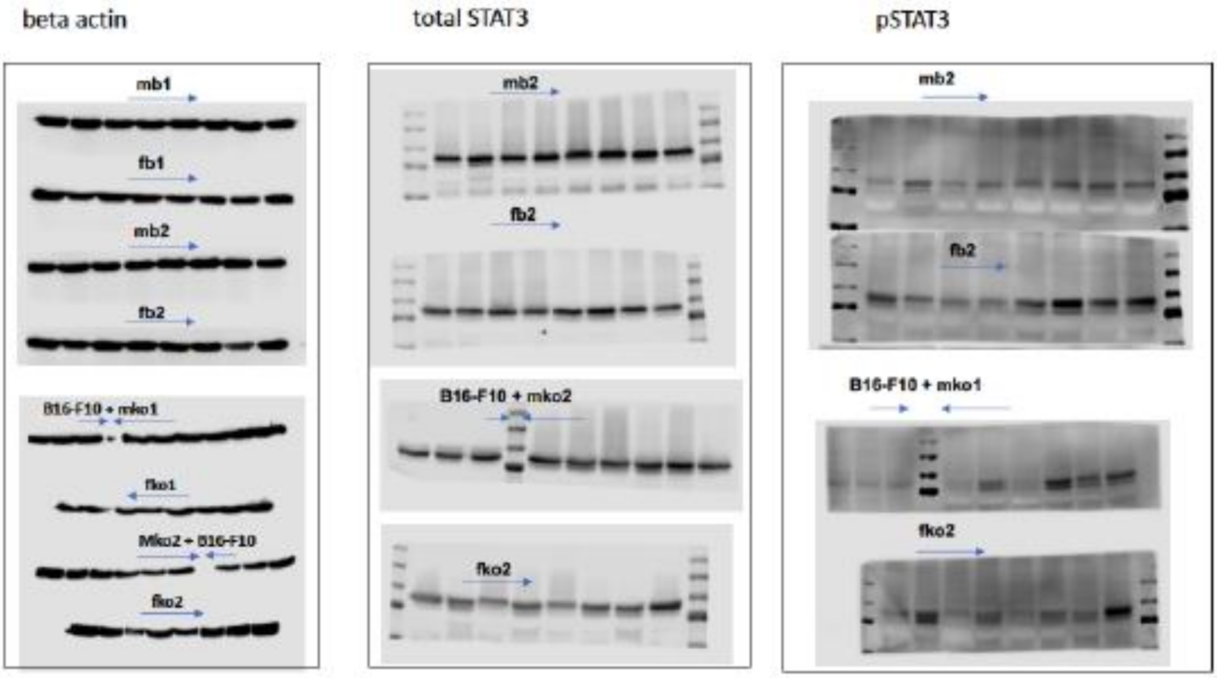

Notes: m-male, f-female, b-tumor proteins from bGH vs WT mice, ko- tumor proteins from GHRKO vs WT, B16-F10- B16-F10 cells in culture. Numbers indicate different gels or blots. Arrow indicates the orientation of sample loading.

Fig. S9. Original images for Western blotting.

Original images for Fig. 2K-L

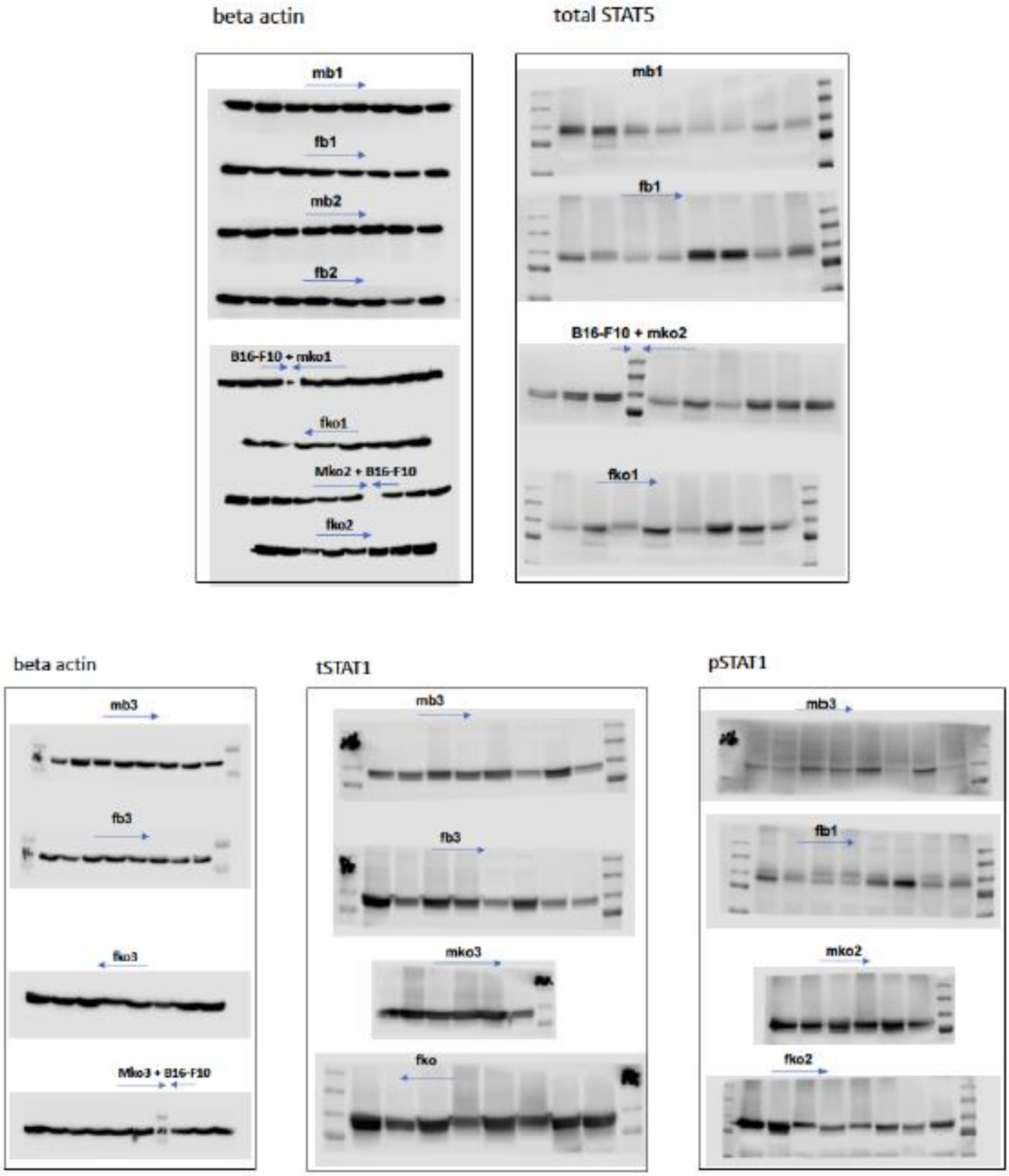

Notes: m-male, f-female, b-tumor proteins from bGH vs WT mice, ko- tumor proteins from GHRKO vs WT, B16-F10- B16-F10 cells in culture. Numbers indicate different gels or blots. Arrow indicates the orientation of sample loading.

Fig. S9. Original images for Western blotting.

Original images for Fig. 2K-L, S5E-F, S6F-G

Fig. 2K-L, S5E-F, S6F-G Female GHRKO vs WT tumors

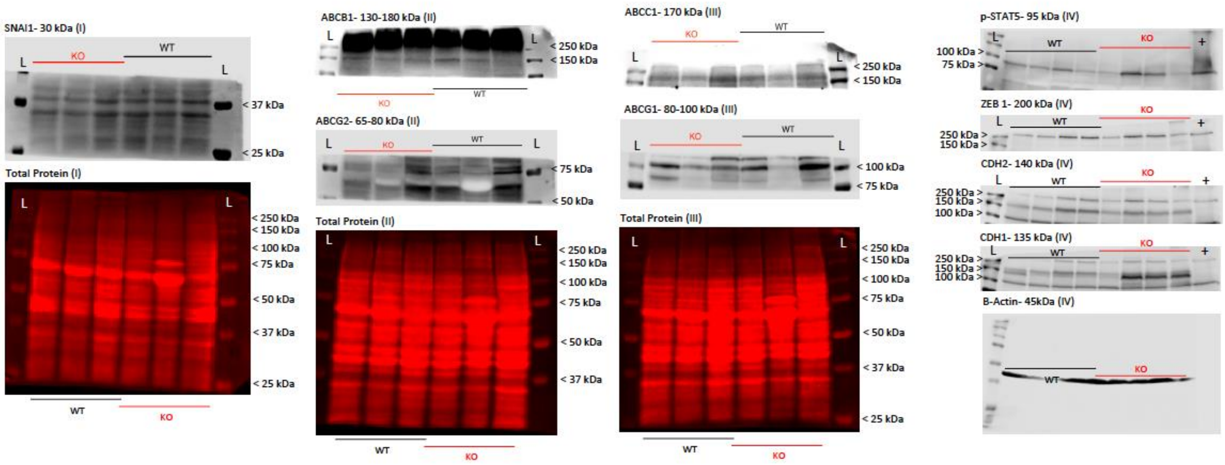

Fig. 2K-L, S5E-F, S6F-G Male GHRKO vs WT tumors

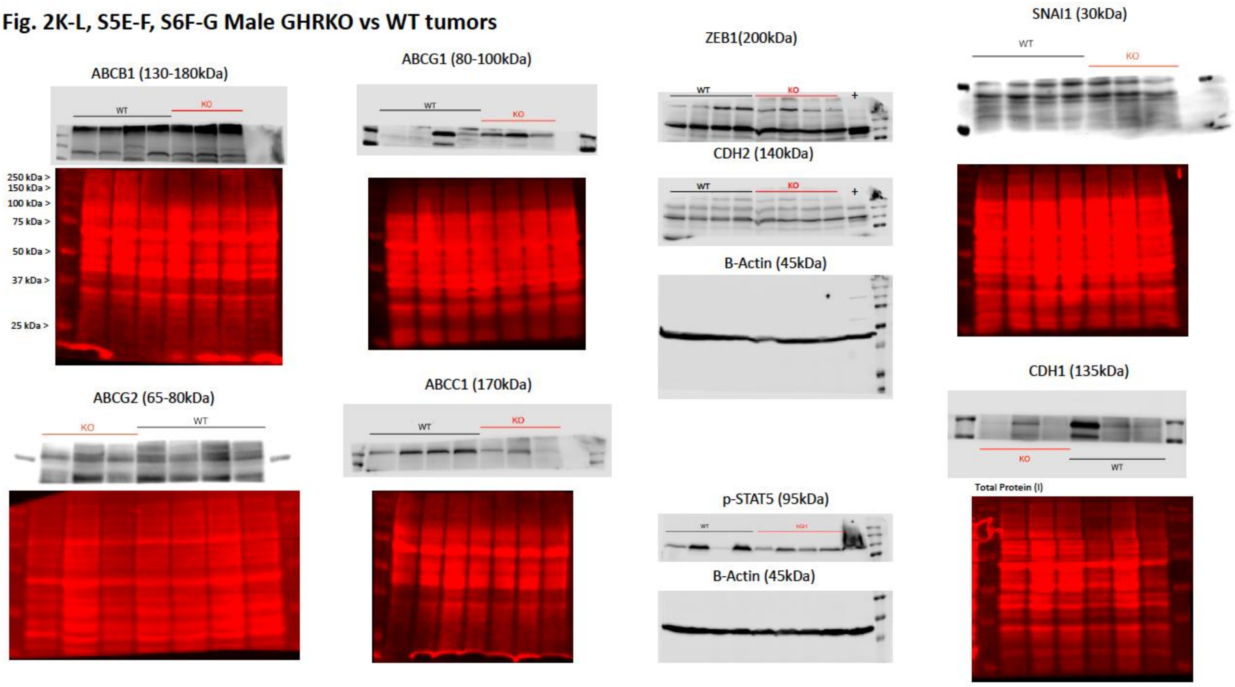

Fig. S9. Original images for Western blotting.

Original images for Fig. 2K-L, S5E-F, S6F-G

Fig. 2K-L, S5E-F, S6F-G Male bGH vs WT tumors

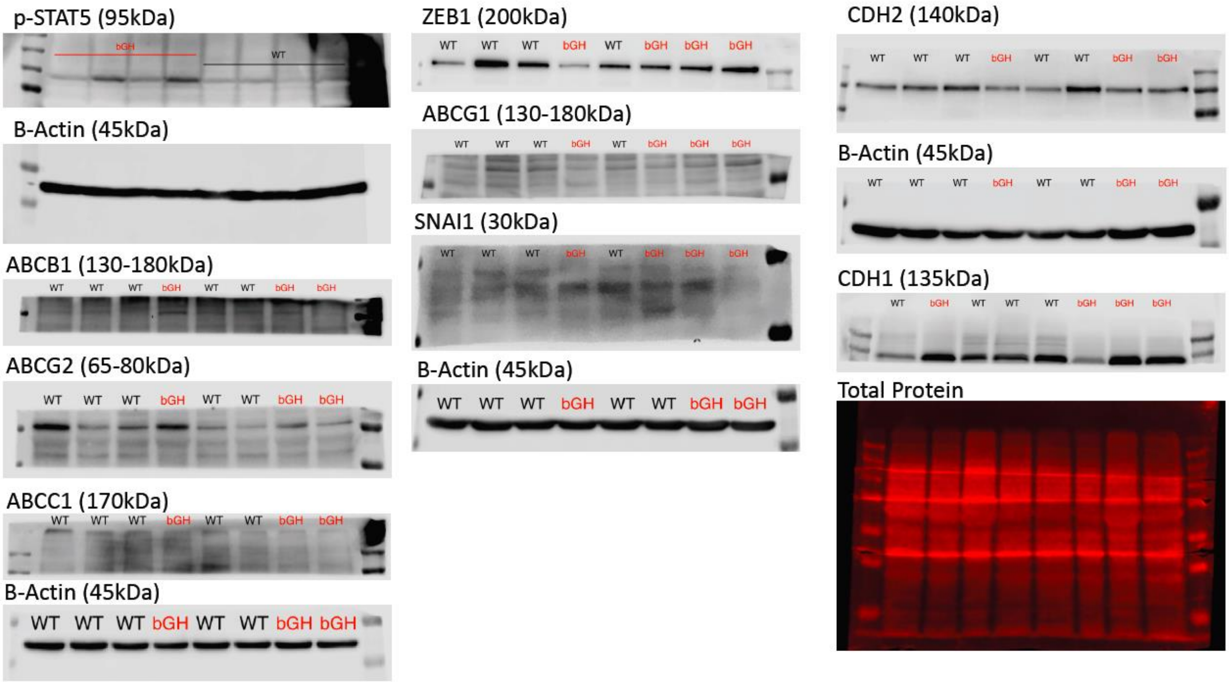

Fig. 2K-L, S5E-F, S6F-G Female bGH vs WT tumors

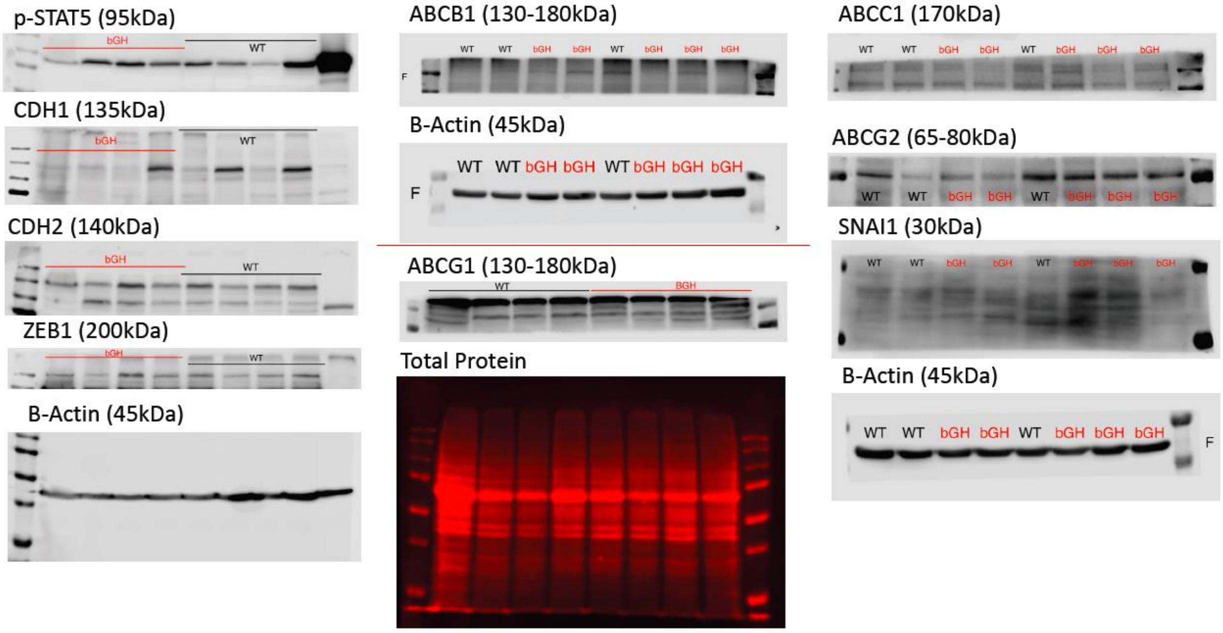

Fig. S9. Original images for Western blotting.

Original images for Fig. 3E-F, 4C-D

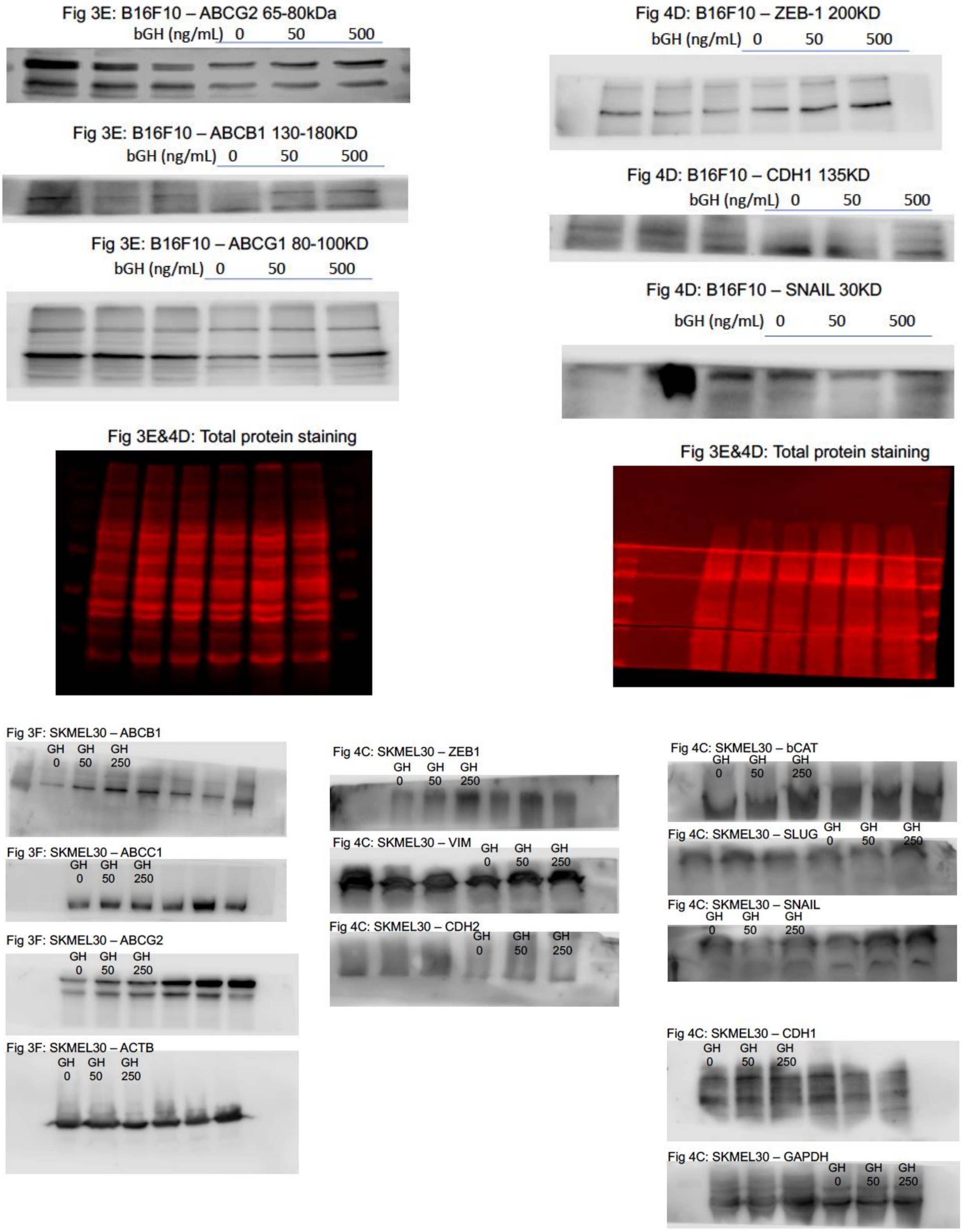

Table S1. Primer sequences for real-time RT-qPCR and siRNA sequences for mouse and human genes.

Gene	Full name	Forward(5'->3')	Reverse(5'->3')
Ghr	Mouse growth hormone receptor	GCCTGGGGACAAGTTCTTCTGGA	TGCAGCTTGTCGTTGGCTTTCCC
Igf-1	Mouse insulin like growth factor 1	GACAAACAAGAAAACGAAGC	ATTTGGTAGGTGTTTCGATG
Gh	Mouse growth hormone	TCCAGTCTGTTTTCTAATGC	TCGAACTCTTTGTAGGTGTC
Igf-1r	Mouse insulin like growth factor 1 receptor	AGAACCGAATCATCATAACG	TTTTAAATGGTGCCTCCTTG
Abcb1a	ATP Binding Cassette Subfamily B Member 1A	AGGACAAAAGAAGGAACTTG	GATAAGGAGAAAAGCTGCAC
Abcb8	ATP Binding Cassette Subfamily B Member 8	GCTGTAAAGCAGAAGAACTG	CCAAGACCATACAGTTGAAAG
Abcc1	ATP Binding Cassette Subfamily C Member 1	GTCTATCGTAAGGCTCTTTTG	GACCAGATCATGTTAATGTACG
Abcc2	ATP Binding Cassette Subfamily C Member 2	CGTATATAAGAAGGCACTAACC	CAATCTGTAAAACACTGGACC
Abcc4	ATP Binding Cassette Subfamily C Member 4	AAACAAAGTCATCCTGTTCG	CAGAAAGTTCTTGATCCTCC
Abcg1	ATP Binding Cassette Subfamily G Member 1	CCAGCTTTATGTCCTAAGTC	CACTTCCATGACAAAGTCTG
Abcg2	ATP Binding Cassette Subfamily G Member 2	AAGAGCCAGTCTATGTTACC	AAACTCCAGCTCTATTTTGC
Zeb1	Zinc finger E-Box binding homeobox 1	GAAACCAGGATGAAAGACAAG	TTCCGAGTTTTCTTTTTGGG
Snai1	Snail family transcription repressor 1	AGTTGACTACCGACCTTG	AAGGTGAACTCCACACAC
Vim	Vimentin	GAACCTGAGAGAACTAACC	GATGCTGAGAAGTCTCATTG
Cdh1	Cadherin-1 (E-cadherin)	CATGTTCACTGTCAATAGGG	GTGTATGTAGGGTAACTCTCTC
Cdh2	Cadherin-2 (N-cadherin)	GAGTTTACTGCCATGACTTTC	TCCACCACTGATTCTGTATG
Gapdh	Glyceraldehyde-3-phosphate dehydrogenase	ACCACAGTCCATGCCATCAC	CACCACCCTGTTGCTGTAGCC
B2m	Beta-2 microglobulin	CTGGTCTTTCTATATCCTGGCT	CATGTCTCGATCCCAGTAGAC
Eif3f	Eukaryotic translation initiation factor 3, subunit F	TACGAACGCCGCAACGAGGG	TGGCACCGAAAAGCAGTTGGTGA
Hprt	Hypoxanthine phosphoribosyltransferase 1	ATCAGTCAACGGGGGACATA	AGAGGTCCTTTTCACCAGCA
siRNA-1	siRNA-1 targeting mouse GHR	CCAGUGUACUCAUUGAGAATT	UUCUCAAUGAGUACACUGGAC
siRNA-2	siRNA-2 targeting mouse GHR	GCCCUAUAUGGUUAACAUATT	UAUGUUAACCAUAUAGGGCCC
siRNA-3	siRNA-3 targeting mouse GHR	GAAAGAATGCCCTGATTAT	
siRNA-B	siRNA targeting human GHR	AGCUAGAAUUGAGUGUUUAAAGUTC	
GH1	Human Growth hormone	AGGAAACACAACAGAAATCC	TTAGGAGGTCATAGACGTTG
GHR	Human Growth hormone receptor	CTCCTCAAGGAAGGAAAATTAG	GTGGAATTCGGGTTTATAGC
IGF1	Human Insulin like growth factor 1	TTATTTCAACAAGCCCACAG	AATGTACTTCCTTCTGGGTC
IGF-1R	Human Insulin like growth factor 1 receptor	AGGGAATTACTCCTTCTACG	TTTATGTCCCCTTTGCTTTG
ABCB1	ATP Binding Cassette Subfamily B Member 1	CGTTGAAGAGTAGAACATGAAG	TTGCACCTCTCTTTTATCTG
ABCB5	ATP Binding Cassette Subfamily B Member 5	AGGATGGTCATCTCATTGAC	CTATGACTGTTCGGATTGATG
ABCB8	ATP Binding Cassette Subfamily B Member 8	GACAAGACATCACCTTCTTTG	GAAGGATGACTTAAACTCCTG
ABCC1	ATP Binding Cassette Subfamily C Member 1	AGCAGAAAAATGTGTTAGGG	TACCCACTGGTAATACTTGG
ABCC2	ATP Binding Cassette Subfamily C Member 2	AAATTGCTGATCTCCTTTGC	GATAGCTGTCCGTACTTTTAC
ABCG1	ATP Binding Cassette Subfamily G Member 1	ATCTCCTATGTCAGGTATGG	AGGGAGATGAAGAAAATCCC
ABCG2	ATP Binding Cassette Subfamily G Member 2	AAAGCCACAGAGATCATAGAG	GATCTTCTTCTTCTTCTCACC
ZEB1	Zinc finger E-Box binding homeobox 1	AAAGATGATGAATGCGAGTC	TCCATTTTCATCATGACCAC
SNAI1	Snail family transcription repressor 1	CTCTAATCCAGAGTTTACCTTC	GACAGAGTCCCAGATGAG
SNAI2	Snail family transcription repressor 2	CAGTGATTATTTCCCCGTATC	CCCCAAAGATGAGGAGTATC
VIM	Vimentin	GGAAACTAATCTGGATTCACTC	CATCTCTAGTTTCAACCGTC
CDH1	Cadherin-1 (E-cadherin)	CCGAGAGCTACACGTTC	TCTTCAAAATTCACTCTGCC
CDH2	Cadherin-2 (N-cadherin)	CTGGAACATATGTGATGACC	TGTAAACATGTTGGGTGAAG
CLDN1	Claudin 1	TTGGCATGAAGTGTATGAAG	ACCTGCAAGAAGAAATATCG
GAPDH	Glyceraldehyde-3-phosphate dehydrogenase	CTTTTGCGTCGCCAG	TTGATGGCAACAATATCCAC

Table S2. Tumor weight and organs from bGH transgenic and WT C57BL/6J mice at dissection

Gender	Genotype	Tumor (g)	Body length (cm)	Lung (g)	Liver (g)	Brain (g)	Heart (g)	Kidney (g)	Spleen (g)
Male	bGH	2.3±0.6	11.1±0.2***	0.24±0.02**	2.99±0.02***	0.46±0.02**	0.29±0.03**	0.52±0.01***	0.40±0.10
	WT	4.8±1.5	9.4±0.02	0.14±0.02	1.30±0.08	0.39±0.01	0.14±0.01	0.32±0.02	0.28±0.07
Famale	bGH	3.3±1.3	10.7±0.1***	0.24±0.02**	2.47±0.15***	0.44±0.04	0.26±0.02***	0.44±0.04**	0.42±0.09
	WT	1.8±0.4	8.9±0.1	0.15±0.01	1.02±0.06	0.37±0.01	0.12±0.01	0.25±0.01	0.20±0.01

Notes: Data are presented as mean ± standard error. bGH group was compared with WT group per gender. 2-tailed unpaired Student's t test, *, $p \leq 0.05$; **, $p \leq 0.01$; ***, $p \leq 0.001$. Male bGH n=5, male WT n=6, female bGH n=7, female WT n=6.

Table S3. Tumor weight and organs from GHRKO and WT C57BL/6J mice at dissection

Gender	Genotype	Tumor (g)	Body length (cm)	Lung (g)	Liver (g)	Brain (g)	Heart (g)	Kidney (g)	Spleen (g)
Male	GHRKO	2.6±0.4	6.4±0.1***	0.08±0.01***	0.42±0.02***	0.36±0.01	0.06±0.01***	0.09±0.01***	0.06±0.02**
	WT	3.2±0.7	9.6±0.1	0.18±0.02	1.45±0.06	0.35±0.01	0.16±0.01	0.36±0.03	0.25±0.04
Famale	GHRKO	2.5±0.7	6.5±0.02***	0.04±0.02**	0.39±0.04***	0.33±0.01*	0.05±0.01***	0.09±0.01***	0.05±0.01*
	WT	1.6±0.5	9.1±0.1	0.15±0.01	1.06±0.03	0.38±0.01	0.15±0.01	0.29±0.01	0.26±0.07

Notes: Data are presented as mean ± standard error. GHRKO group was compared with WT group per gender. 2-tailed unpaired Student's t test, *, $p \leq 0.05$; **, $p \leq 0.01$; ***, $p \leq 0.001$. Male GHRKO n=6, male WT n=8, female GHRKO n=4, female WT n=5.
